# Combined exposure to emerging *Fusarium* mycotoxins produces concentration-dependent synergistic toxicity in Jurkat T cells

**DOI:** 10.3389/ftox.2026.1924250

**Published:** 2026-09-23

**Authors:** Chanamon Maleeratana, Phattarawadee Wattanasuntorn, Saranya Poapolathep, Imourana Alassane-Kpembi, Johanna Fink-Gremmels, Isabelle P. Oswald, Amnart Poapolathep

**Affiliations:** 1 Department of Pharmacology, Faculty of Veterinary Medicine, Kasetsart University, Bangkok, Thailand; 2 Department of Veterinary Biomedicine, Faculty of Veterinary Medicine, Université de Montréal, Saint-Hyacinthe, QC, Canada; 3 Institute for Risk Assessment Sciences, Faculty of Veterinary Medicine, Utrecht University, Utrecht, Netherlands; 4 Toxalim (Research Centre in Food Toxicology), Toulouse University, INRAE, ENVT, INP-Purpan, UPS, Toulouse, France

**Keywords:** beauvericin, enniatin B, moniliformin, cytotoxicity, interaction, human Jurkat T cells

## Abstract

**Introduction:**

Beauvericin (BEA), enniatin B (ENN B), and moniliformin (MON), mainly produced by Fusarium species, are often described as emerging mycotoxins due to their frequent detection in cereals and cereal‐based products and their current lack of regulatory limits. Despite increasing evidence regarding their individual toxicities–including immunotoxic, genotoxic, reproductive, and cardiotoxic effects–information on their combined toxicity and the underlying mechanisms remains limited. To address this gap, this study aimed to evaluate the cytotoxic effects, reactive oxygen species (ROS) generation, and interaction patterns of BEA, ENN B, and MON in human Jurkat T cells following a 24‐hour exposure under individual, dual, and triple treatment conditions.

**Methods:**

The experimental approach evaluated cellular responses and toxicological interactions across single and combined exposures. Cytotoxicity was assessed using the MTT assay to determine cell viability, while intracellular ROS levels were measured using a fluorescence‐based assay. To analyze the dual and mixture interaction patterns among the mycotoxins, the study utilized the Chou–Talalay method for quantitative assessment of synergism and antagonism.

**Results:**

The individual toxicity assessments yielded IC_50_ values of 1.81 μM for BEA, 4.47 μM for ENN B, and 40.45 μM for MON, establishing the potency ranking of BEA > ENN B > MON. Across all treatments, cell viability decreased in a concentration-dependent manner, which was consistently accompanied by an elevation in intracellular ROS fluorescence signals. Combination Index (CI) analysis of binary mixtures (BEA + ENN B, BEA + MON, and ENN B + MON) revealed synergistic interactions at low to moderate effect levels (IC_10_–IC_50_), which shifted toward antagonism at higher effect levels (IC_75_–IC_90_). In contrast, the ternary mixture of all three mycotoxins (BEA + ENN B + MON) predominantly exhibited sustained synergism across the entire evaluated concentration range (IC_10_–IC_90_).

**Discussion:**

Overall, these findings demonstrate that co-exposure to BEA, ENN B, and MON leads to complex interactive toxicities, with the ternary mixture showing persistent synergistic effects based on Combination Index analysis. Although these cellular responses strongly correlate with elevated ROS‐associated fluorescence signals, direct experimental validation is still required to confirm the specific downstream molecular mechanisms involved.

## 1 Introduction

Mycotoxin contamination of food and feed remains a persistent global food safety challenge, particularly in cereal-based commodities that constitute a major component of the human diet (Yilmaz et al., 2025; Di Salvo et al., 2025). Cereals such as maize, wheat, barley, and rice are highly vulnerable to fungal invasion both in the field and during storage, creating multiple opportunities for toxin production along the supply chain (Niaz et al., 2025; Fărcaş, 2024). Among toxigenic fungi, species of the genus *Fusarium* are of particular concern due to their widespread distribution in agricultural environments, adaptability to diverse climatic conditions, and capacity to produce a diverse spectrum of secondary metabolites (Bugingo et al., 2025; Wadhwa et al., 2024). While regulatory attention has historically focused on well-known *Fusarium* mycotoxins such as deoxynivalenol, fumonisins, and zearalenone, increasing analytical sensitivity in recent years has revealed frequent contamination by so-called emerging mycotoxins (Kanabus et al., 2026; Chilenga et al., 2025). These compounds, including beauvericin (BEA), enniatin B (ENN B), and moniliformin (MON), are not yet comprehensively regulated by the competent authorities despite accumulating evidence of their biological activity and frequent occurrence in food chains (Behr et al., 2025; Moyano-López et al., 2025).

BEA and ENN B belong to the group of cyclic hexadepsipeptides, whereas MON is a small, highly polar mycotoxin with a distinct chemical structure (Zhang et al., 2025; Mischler et al., 2024). All three are primarily produced by various *Fusarium* species commonly associated with cereal crops such as maize, wheat, barley, and rice (Sun et al., 2025; Niaz et al., 2025). Advances in liquid chromatography–high resolution mass spectrometry (LC-HRMS) and multi-residue analytical methods have demonstrated that these *Fusarium* mycotoxins are widely present in raw grains, processed cereal products, and animal feeds across Europe, Asia, and other regions (Maphaisa et al., 2025; Aghzaf et al., 2025). Importantly, biomonitoring and occurrence studies consistently report the co-presence of BEA, ENN B, and MON within the same samples, reflecting shared fungal sources and overlapping ecological niches (Shen et al., 2025; Bosch et al., 2023). From an exposure assessment perspective, this constant co-occurrence suggests that consumers and livestock are more likely to encounter these toxins as mixtures rather than as individual contaminants (Vilela et al., 2025). Toxicological investigations over the past decade have begun to elucidate the biological effects of these emerging Fusarium mycotoxins (Qu et al., 2024). BEA and ENN B are well recognized for their ionophoric properties, enabling disruption of cellular ion homeostasis, mitochondrial dysfunction, oxidative stress induction, and apoptosis (Coulet et al., 2025; Søderstrøm et al., 2022). MON, although structurally distinct, has been associated with interference in energy metabolism, inhibition of key enzymatic pathways, and potential cardiotoxic and immunotoxic effects (Sharma et al., 2012).

Despite this growing body of knowledge, current food safety risk assessments largely rely on data derived from single-compound exposure. This reductionist approach does not adequately reflect real-world exposure scenarios (Ball et al., 2022). In practice, through contaminated diets humans and animals are chronically exposed to complex mixtures of toxicants including (Alassane-Kpembi et al., 2013; Kolf-Clauw et al., 2013; Garofalo et al., 2023). Surveys of cereals and cereal-based products repeatedly demonstrate simultaneous occurrence of multiple Fusarium metabolites, including BEA, ENN B, and MON (Fremy et al., 2019). The potential for additive, synergistic, or antagonistic interactions among co-occurring mycotoxins therefore represents a critical but insufficiently characterized aspect in food safety assessment (Gruber-Dorninger et al., 2017). Mixture toxicology has increasingly emphasized that combined exposures may produce effects that cannot be predicted from individual compounds alone (Ribeiro et al., 2017). Synergistic interactions, in particular, may amplify toxicity even when each mycotoxin is present at sub-toxic levels, potentially leading to underestimation of risk under current assessment paradigms (Zhu et al., 2025). Conversely, antagonistic interactions may obscure hazard signals and complicate interpretation of exposure data (Kolf-Clauw et al., 2013; Willoquet et al., 2024). Regulatory bodies and international food safety authorities have repeatedly highlighted the need for mixture-based risk assessment frameworks (D’Amore et al., 2025). However, experimental evidence to support such approaches remains limited for many emerging mycotoxins.

Synergism can be expected when toxins are structurally related and have the same biological activity, such as BEA, ENN B. Moreover, oxidative stress has emerged as a central mechanism in mycotoxin-induced cellular injury (Szőke et al., 2025). Elevated intracellular reactive oxygen species (ROS) have been reported following exposure to BEA and ENN B, and growing evidence suggests that MON may also contribute to redox imbalance (Juan et al., 2021). Evaluating ROS generation alongside cytotoxicity is therefore a valuable additional quantifiable parameter in the assessment of mixture toxicity (Snezhkina et al., 2019).

The immune system represents a biologically plausible and sensitive target for dietary mycotoxin exposure. Immune cells are highly responsive to redox perturbations, membrane-active compounds, and mitochondrial stress (Kraft et al., 2021). Chronic exposure to contaminated food has been associated with altered immune function and increased susceptibility to disease in both humans and animals. Human T lymphocytes, which are central to adaptive immunity, may be particularly vulnerable to such insults (Chałaśkiewicz et al., 2025; Benkerroum, 2020). The human Jurkat T-cell line has been widely used as an *in vitro* model for studying T-cell signaling, apoptosis, and xenobiotic-induced cytotoxicity (Wattanasuntorn et al., 2025; Wilson et al., 2025). Toxicological interactions are often dose-dependent, and conclusions drawn from a single effect level may be misleading. The Chou–Talalay method, originally developed to measure the interactions between pharmacologically active compounds, provides a robust quantitative framework for distinguishing synergism, additivity, and antagonism through calculation of the combination index (CI) and dose‐reduction index (DRI) (Chou, 2010; Ashton, 2015). Generating interaction data across multiple fractional effect levels (e.g., IC_10_–IC_90_) is therefore valuable for the prediction of adverse effects of mixture in risk assessment.

Hence the objective of the current study was to present experimental evidence for the interaction of the BEA, ENN B and MON in a Jurkat cell model using cytotoxicity and ROS generation as common endpoints.

## 2 Materials and methods

### 2.1 Chemicals and reagents

BEA (C_45_H_57_N_3_O_9_, 783.9 g/mol), ENN B (C_33_H_57_N_3_O_9_, 639.8 g/mol), and MON (C_4_H_2_O_3_, 98.06 g/mol) analytical standards (purity ≥98%) were purchased from Sigma-Aldrich (Darmstadt, Germany). Dimethyl sulfoxide (DMSO), phosphate-buffered saline (PBS), and all other analytical-grade reagents were obtained from Sigma-Aldrich unless otherwise specified. Cell culture reagents including RPMI-1640 medium, fetal bovine serum (FBS), penicillin–streptomycin solution, and trypsin-free cell dissociation buffer were purchased from Gibco (Thermo Fisher Scientific, Waltham, MA, United States). The MTT reagent (3-(4,5-dimethylthiazol-2-yl)-2,5-diphenyltetrazolium bromide) used for cell viability assessment was obtained from Sigma-Aldrich. The Total ROS Assay Kit used for intracellular reactive oxygen species measurement was obtained from Solarbio Life Sciences (catalog number: CA1410; Beijing, China).

### 2.2 Cell culture

Human Jurkat T lymphocyte cells (catalog number: EP-CL-0129; Elabscience, Houston, TX, United States) were used as an experimental model in this study. To maintain biological consistency and minimize variations across replicates, all experiments were strictly conducted using Jurkat T cells passages 8 to 12 (P8 – P12). Cells were seeded at a standard density of 1 × 10^4^ cells per well in 100 µL of complete culture medium prior to treatment. Cells were cultured in RPMI-1640 medium supplemented with 10% fetal bovine serum (FBS) and 1% penicillin–streptomycin. The cultures were maintained in a humidified incubator at 37 °C under an atmosphere of 5% CO_2_. Culture medium was refreshed every 2 days to ensure optimal growth conditions.

For toxicity experiments, cells were seeded into 96-well plates at a density of 1 × 10^4^ cells per well in 100 µL of culture medium and allowed to stabilize prior to treatment. Subsequently, the cells were exposed to different concentrations of individual mycotoxins as well as their combinations to evaluate both single-compound and mixture effects.

### 2.3 Experimental design and toxin exposure

BEA, ENN B, and MON were first evaluated individually to determine their cytotoxic effects using five serial two-fold dilutions. Jurkat T cells were exposed to BEA at concentrations ranging from 0.45 to 7.24 µM, ENN B from 1.12 to 17.88 µM, and MON from 10.11 to 161.8 µM. The concentration ranges were determined based on preliminary dose–response experiments to capture a broad spectrum of biological activity, including sub-toxic, half-maximal inhibitory (IC_50_), and highly cytotoxic levels. In parallel, rigorous experimental controls were included in every 96-well plate for quality assurance: Blank Control: Culture medium without cells was included to subtract background absorbance. Negative (Untreated) Control: Intact cells cultured in complete medium without toxins were included for quality assurance to monitor baseline cell growth. Vehicle Control: Cells were exposed to vehicle control matching the highest final DMSO concentration (<0.1% v/v) and served as the standard 100% cell viability reference baseline for data normalization.

To investigate potential interaction effects among the mycotoxins, binary and ternary mixtures were prepared using an equipotent constant-ratio design, which maintains a fixed proportion of each compound across the dilution series. This approach allows for systematic evaluation of mixture toxicity and interaction patterns. Similar to the individual toxin experiments, each mixture treatment was tested using five serial two-fold dilutions, with untreated and vehicle control groups included under the same experimental conditions.

For binary combinations, three toxin pairs were examined: BEA + ENN B, BEA + MON, and ENN B+ MON. The concentration ranges for these mixtures were 0.45–7.24 µM + 1.12–17.88 µM, 0.45–7.24 µM + 10.11–161.8 µM, and 1.12–17.88 µM + 10.11–161.8 µM, respectively. In addition, a ternary combination consisting of BEA, ENN B, and MON was tested using the same equipotent constant-ratio principle across five two-fold serial dilutions, covering concentration ranges of 0.45–7.24 µM, 1.12–17.88 µM, and 10.11–161.8 µM, respectively. Each treatment condition was performed in triplicate wells and repeated in at least three independent experiments.

Fixed-ratio combinations were designed based on the equipotent IC_50_ values of individual mycotoxins determined in 24 h cytotoxicity assays in Jurkat T cells (BEA = 1.81 µM, ENN B = 4.47 µM, and MON = 40.45 µM; Table 1). Specifically, the fixed molar ratios were calculated directly from these experimentally fitted IC_50_ values as follows: BEA: ENN B (1:2.47, derived from 1.81:4.47 µM), BEA: MON (1:22.35, derived from 1.81:40.45 µM), ENN B: MON (1:9.05, derived from 4.47:40.45 µM), and BEA: ENN B: MON (1:2.47:22.35, derived from 1.81:4.47:40.45 µM). Dose–response parameters (*Dm, m, r*), Combination Index (CI), and dose‐reduction index (DRI) values were calculated separately for each of three independent biological experiments (n = 3) using CompuSyn software (version 1.0; ComboSyn Inc., Paramus, NJ, United States). Results are presented as mean ± standard deviation (SD).

**TABLE 1:** Cytotoxicity of BEA, ENN B and MON in cultured human Jurkat T cells (1 × 10^4^ cells/well), based on IC_50_ value, determined using MTT assay. Cells were treated for 24 h with tested mycotoxins at variable concentrations.

Mycotoxins	IC_50_ (µM)
BEA	1.81
ENN B	4.47
MON	40.45

### 2.4 Cell viability assay

Cell viability was determined using the MTT colorimetric assay. Jurkat T cells (passages 8–12) were seeded into 96-well plates at a density of $1\times 10^{4}$ cells per well in 100 µL of complete culture medium. After seeding, the cells were treated with 100 µL per well of 2× concentrated mycotoxin solutions under individual, binary, and ternary exposure conditions at the concentrations described above (yielding a final working volume of 200 µL per well) and incubated for 24 h under standard culture conditions. All mycotoxin concentrations reported in this study correspond to final in-well working concentrations. Experimental plates included blank wells (medium with MTT without cells), untreated control cells (cells with culture medium only), and vehicle control cells (treated with 0.1% DMSO, matching the highest final vehicle concentration used in treatment wells). No chemical positive cytotoxicity control was included in the assay routine. Following the exposure period, 10 µL of MTT solution (3-(4,5-dimethylthiazol-2-yl)-2,5-diphenyltetrazolium bromide) at a concentration of 5 mg/mL was added to each well. The plates were then incubated at 37 °C for 4 h to allow metabolically active cells to reduce the yellow tetrazolium salt to insoluble purple formazan crystals. After incubation, 100 µL of dimethyl sulfoxide (DMSO) was added to each well to dissolve the formed formazan crystals, and the plates were gently agitated on a shaker for 30 min to ensure complete solubilization. The absorbance (optical density, OD) was measured at 540 nm using a microplate reader (Multiskan GO, Thermo Fisher Scientific, Waltham, MA, United States). Absorbance values were background-corrected using the blank wells, and cell viability was expressed as a percentage relative to the vehicle control cells (0.1% DMSO), according to the equation shown below:

$$\text{Cell viability }\left(\%\right)=100\;x\;\frac{\text{mean}\;\text{OD}\;\text{of}\;\text{mycotoxin}\;\text{treated}\;\text{samples}}{\text{mean}\;\text{OD}\;\text{of}\;\text{vehicle}\;\text{treated}\;\text{samples}}$$

### 2.5 Analysis of mycotoxin interactions

The interactions among BEA, ENN B, and MON were evaluated across different fractional effect levels (fa) using the Chou–Talalay method. Interaction analysis was specifically focused on low fractional-effect levels (IC_10_ –IC_50_) within the experimental dose–response curves to determine interaction patterns under sub-maximal cytotoxic conditions, rather than at high inhibitory effect levels (Alassane Kpembi et al., 2017). It should be noted that these fractional effect levels describe relative cell inhibition *in vitro* and do not directly equate to physiologically relevant or realistic human dietary exposure concentrations. In addition, the use of low doses allows for the identification of potential synergistic interactions that may not be apparent at higher concentrations.

The fraction affected (Fa) values derived from the MTT cell viability assay were used as input parameters for the analysis of combination effects. The relationship between dose and effect was first described using the median–effect principle. The corresponding median–effect equation used for the calculation is presented below:

$$\frac{f_{a}}{f_{u}}={\left(\frac{D}{D_{m}}\right)}^{m}$$

In this equation, *fa* represents the fraction affected, while *fu* denotes the fraction unaffected (*fu =* 1 *– fa)*. D corresponds to the dose of the compound, D_m_ represents the median-effect dose (i.e., the dose required to produce a 50% effect), and m is the slope of the median-effect plot (Hill-type coefficient), which describes the shape of the dose–response curve. Values of m < 1, m = 1, and m > 1 indicate flat sigmoidal, hyperbolic, and sigmoidal dose–effect curves, respectively.

Based on the median–effect parameters, the combination index (CI) was calculated to determine the nature of the interactions between the tested mycotoxins. The general equation used for CI determination is shown below:

$$CI=\frac{{\left(D\right)}_{1}}{{\left(D_{x}\right)}_{1}}+\frac{{\left(D\right)}_{2}}{{\left(D_{x}\right)}_{2}}$$

In this equation (D)_1_ and (D)_2_ represent the doses of each mycotoxin in the combination required to produce x% inhibition. In contrast (D_x_)_1_ and (D_x_)_2_ correspond to the doses of each mycotoxin administered individually that produce the same x% inhibitory effect. The nature of interactions among BEA, ENN B, and MON was quantitatively evaluated using the Combination Index (CI) method based on the median-effect principle of Chou and Talalay (Chou, 2006). To account for biological variability near pure additivity (CI = 1.0) and to prevent the overstatement of interaction strength, combination effects were systematically categorized using the standard graded Chou–Talalay terminology framework:

CI < 0.10: Very strong synergism CI = 0.10–0.30: Strong synergism.

CI = 0.30–0.70: Synergism CI = 0.70–0.85: Moderate synergism.

CI = 0.85–0.90: Slight synergism CI = 0.90–1.10: Nearly additive/Additivity.

CI = 1.10–1.20: Slight antagonism CI = 1.20–1.45: Moderate antagonism.

CI = 1.45–3.30: Antagonism CI > 3.30: Strong to very strong antagonism.

Dose combinations yielding CI values within the range of 0.90−1.10 were defined as nearly additive to accommodate inherent experimental noise in cell-based assays. All CI values were calculated across specific fractional effect levels ( fa=0.10−0.90 , corresponding to $IC_{10}-IC_{90}$ ) using CompuSyn software (ComboSyn, Inc., Paramus, NJ, United States).

In addition, the dose‐reduction index (DRI) was calculated to estimate the extent to which the dose of each toxin could theoretically be reduced when applied in combination while achieving a comparable biological effect. The equation used for DRI calculation is provided below:

$${\left(DRI\right)}_{1}=\frac{{\left(D_{x}\right)}_{1}}{{\left(D\right)}_{1}},{\left(DRI\right)}_{2}=\frac{{\left(D_{x}\right)}_{2}}{{\left(D\right)}_{2}},.\;.\;.\;etc.$$

Therefore,

$$CI=\frac{{\left(D\right)}_{1}}{{\left(D_{x}\right)}_{1}}+\frac{{\left(D\right)}_{2}}{{\left(D_{x}\right)}_{2}}+...=\frac{1}{{\left(DRI\right)}_{1}}+\frac{1}{{\left(DRI\right)}_{2}}+...$$

All calculations were performed using CompuSyn software (ComboSyn Inc., Paramus, NJ, United States).

### 2.6 Measurement of intracellular reactive oxygen species (ROS)

Intracellular reactive oxygen species (ROS) levels were determined using the Total ROS Assay Kit (catalog number: CA1410; Solarbio Life Sciences, Beijing, China) according to the manufacturer’s instructions. Human Jurkat T cells (passages 8–12, $1\times 10^{4}$ cells per well in 100 µL of complete culture medium) were seeded in black 96-well plates and exposed to increasing concentrations of BEA ( 0−7.24 μM ), ENN B ( 0−17.88 μM ), and MON ( 0−161.80 μM ) by adding 100 µL per well of 2× concentrated mycotoxin solutions to achieve a final working volume of 200 µL per well. Cells were incubated for 24 h under standard culture conditions. All mycotoxin concentrations stated in this study correspond to final in-well working concentrations.

Following the 24 h treatment, cells were harvested and washed twice with cold phosphate-buffered saline (PBS, pH 7.4) via centrifugation at 300×g for 5 min to remove residual toxins and culture medium. Cells were then resuspended and incubated with 10 μM DCFH-DA probe solution (diluted 1:1000 in serum-free medium) at 37 °C in the dark for 30 min. After incubation, cells were washed three times with serum-free medium to thoroughly remove extracellular probe. Fluorescence intensity was measured using a fluorescence microplate reader (Varioskan LUX, Thermo Fisher Scientific, Waltham, MA, United States) set at an exact excitation wavelength of 488 nm and emission wavelength of 525 nm (bandwidth: 12 nm). Blank wells (medium with ROS reagent without cells), untreated control cells, and vehicle control cells (treated with 0.1% v/v DMSO, matching the exact final vehicle concentration across all toxin-treated wells) were included to establish background levels and baseline ROS production. All assays were performed with three technical replicates per condition across three independent biological experiments ( n=3 ). ROS levels were expressed as relative fluorescence units (RFU) relative to untreated control cells.

$$ROS\;\left(RFU\right)=mean\;fluorescence\;intensity\;of\;treated\;samples-mean\;fluorescence\;intensity\;of\;blank$$

### 2.7 Statistical analysis

To ensure high experimental reproducibility, all assays were performed using three independent biological experiments ( n=3 ), with each experiment conducted in technical triplicates. The mean value of technical replicates within each plate served as the statistical unit for each independent run. All data, including cell viability, intracellular ROS levels (expressed as relative fluorescence units, RFU, relative to the vehicle control), derived Combination Index (CI), and median-effect parameters, are presented as the Mean ± standard deviation (SD) across independent experiments. Individual values from the three independent biological runs are overlayed on the respective figures to provide full transparency of data variability.

Differences among multiple treatment groups relative to the vehicle control were evaluated using one-way analysis of variance (ANOVA) followed by Tukey’s Honest Significant Difference (HSD) *post hoc* test. Given the sample size ( n=3 ), parametric statistical assumptions were assumed based on standard toxicological experimental designs and assessed where feasible, though formal normality and variance tests were interpreted with caution. A p -value <0.05 was considered statistically significant (* p<0.05 ). All statistical analyses and data visualizations were performed using GraphPad Prism (version 9.5.1, GraphPad Software, San Diego, CA, United States).

## 3 Results

### 3.1 Effects of BEA, ENN B and MON on cell viability

The effects of BEA, ENN B, and MON on the viability of human Jurkat T cells were evaluated using the MTT assay after 24 h of exposure (Figure 1). Cells were treated with increasing concentrations of BEA (0–7.24 μM), ENN B (0–17.88 μM), and MON (0–161.80 μM). All three mycotoxins induced a concentration-dependent decrease in cell viability. BEA caused a pronounced reduction, with marked cytotoxicity observed at the highest concentration (7.24 μM). ENN B similarly exhibited strong dose-dependent effects, particularly at concentrations above 4.47 μM. In contrast, MON also reduced cell viability but required higher concentrations to achieve comparable effects, with significant cytotoxicity observed at 161.80 μM. Statistically significant differences compared to the control group were detected at multiple concentrations (*p < 0.05). Overall, BEA and ENN B demonstrated greater cytotoxic potency than MON in Jurkat T cells.

**FIGURE 1:**
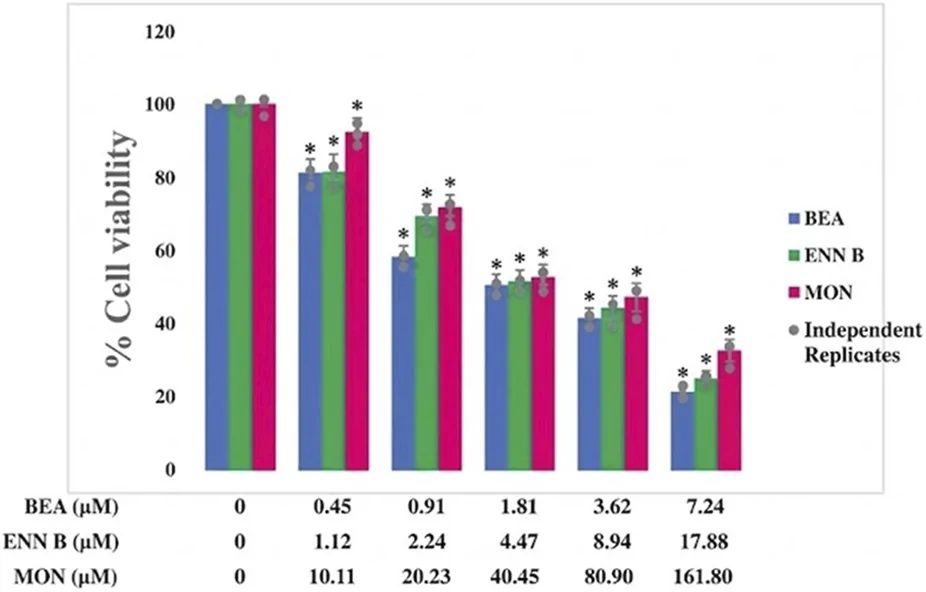
Effects of beauvericin (BEA), enniatin B (ENN B), and moniliformin (MON) on cell viability in human Jurkat T cells (clone E6.1). Exponentially growing Jurkat T cells (1 × 10^4^ cells/well) were treated with increasing concentrations of BEA (0–7.24 μM), ENN B (0–17.88 μM), and MON (0–161.80 μM) for 24 h. Cell viability was assessed by the MTT assay, reflecting the enzymatic reduction of tetrazolium dye in metabolically active cells. Data represent Mean ± SD of three independent biological experiments (n = 3), with individual experimental values shown as overlayed data points. Asterisks (*) denote statistically significant differences compared to the vehicle control group (*p* < 0.05).

The inhibitory concentrations required to achieve 50% growth inhibition (IC_50_) are presented in Table 1. These values were determined from dose–response curves obtained using the MTT assay following 24 h exposure of human Jurkat T cells to the tested mycotoxins. Among the compounds, beauvericin (BEA) exhibited the lowest IC_50_ value (1.81 μM), indicating the highest cytotoxic potency, followed by enniatin B (ENN B) with an IC_50_ of 4.47 μM. In contrast, moniliformin (MON) showed a substantially higher IC_50_ value (40.45 μM), reflecting lower cytotoxicity. Overall, the results demonstrate a clear difference in potency among the tested mycotoxins, with BEA and ENN B exhibiting higher cytotoxic potency in Jurkat T cells than MON based on their lower IC_50_ values.

### 3.2 Combined cytotoxicity of BEA, ENN B and MON on Human Jurkat T cells

The combined effects of BEA, ENN B, and MON on the viability of human Jurkat T cells are presented in Figure 2 (A), co-exposure to BEA and ENN B resulted in lower cell viability compared to individual treatments, particularly at higher concentrations. Similarly, in (B), the combination of BEA and MON showed a more pronounced decrease in cell viability than either compound alone, although the effect was less marked than that observed with BEA and ENN B. In (C), co-treatment with ENN B and MON also led to a concentration-dependent decline in cell viability, with combination effects generally exceeding those of single exposures. Across all combinations, cell viability decreased progressively with increasing concentrations, and several conditions showed statistically significant differences compared to controls (*p < 0.05). Overall, these results suggest that combined exposure to these mycotoxins enhances cytotoxicity in Jurkat T cells, with the BEA and ENN B combination resulting in the lowest observed cell viability values.

**FIGURE 2:**
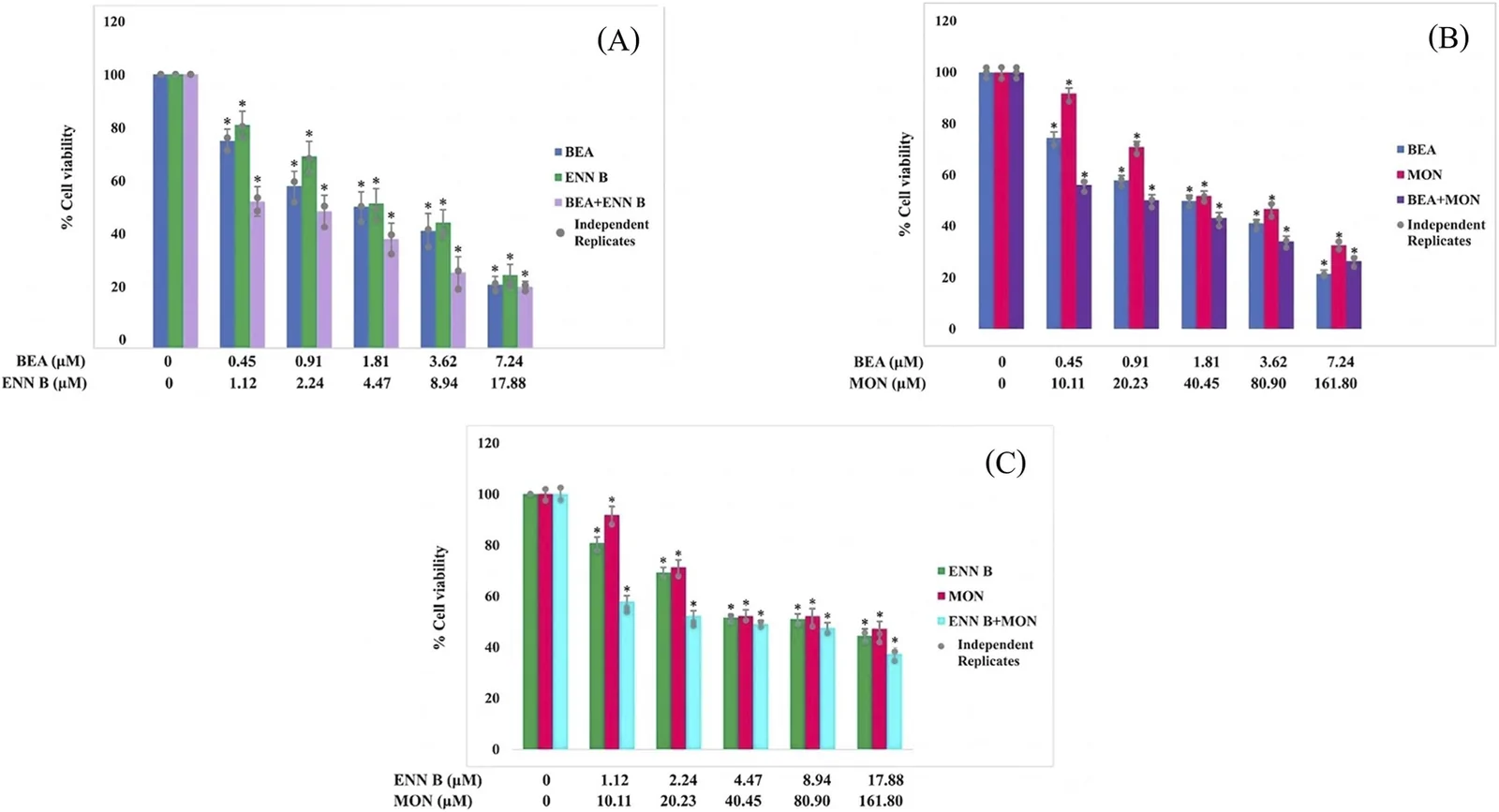
Comparative cytotoxic effects of combined mycotoxin treatments on human Jurkat T cells. Jurkat T cells were exposed to BEA in combination with ENN B **(A)**, BEA with MON **(B)**, and ENN B with MON **(C)** at the indicated concentrations for 24 h. Cell viability was determined using the MTT assay. All combinations induced a concentration-dependent decrease in cell viability, with combined treatments generally exhibiting greater cytotoxic effects than individual compounds. Data represent Mean ± SD of three independent biological experiments (n = 3), with individual experimental values shown as overlayed data points. Asterisks (*) denote statistically significant differences compared to the vehicle control group (*p* < 0.05).

Consistent with these observations, the dose–effect relationship curves for the cytotoxicity of individual mycotoxins and their combinations are presented in Figures 2, 3. Overall, exposure to BEA, ENN B, and MON resulted in a significant, concentration-dependent decrease in cell viability. Descriptively, combined treatments demonstrated lower cell viability percentages across tested concentration ranges compared to single-compound exposures. At all tested concentrations, cell viability following exposure to binary mixtures (BEA + ENN B, BEA + MON, and ENN B+ MON) and the ternary mixture (BEA + ENN B+ MON) was consistently lower than that observed for individual toxins. This trend became more pronounced at higher concentrations, indicating increased toxicity with co-exposure. These findings suggest potential additive or synergistic interactions among the mycotoxins, leading to greater impairment of Jurkat T cell viability compared to single-agent treatments.

**FIGURE 3:**
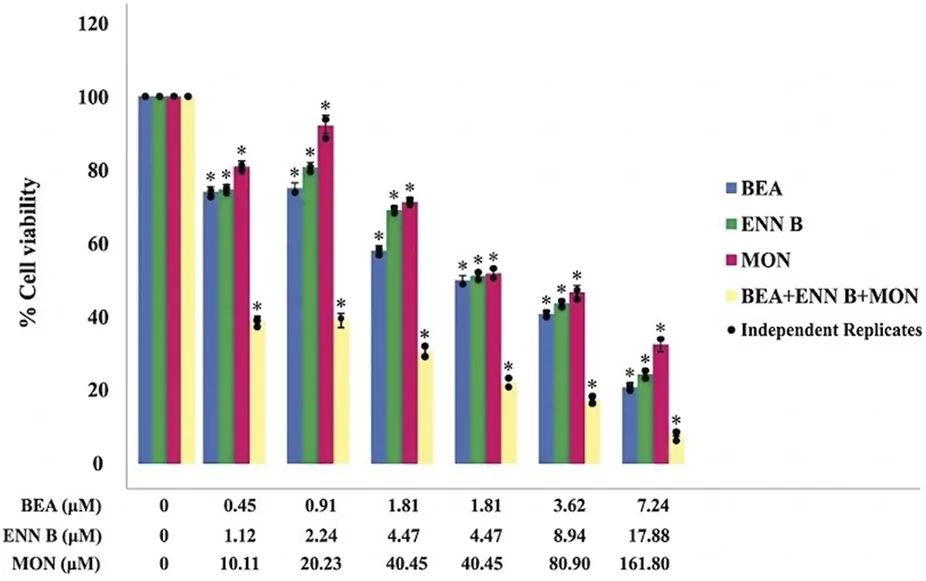
Combined cytotoxic effects of BEA, ENN B, and MON on human Jurkat T cells. Jurkat T cells were treated with BEA, ENN B, and MON individually and in combination (BEA + ENN B+ MON) at the indicated concentrations for 24 h. Cell viability was assessed using the MTT assay. The combined treatment exhibited a more pronounced reduction in cell viability compared to individual mycotoxins. Data represent Mean ± SD of three independent biological experiments (n = 3), with individual experimental values shown as overlayed data points. Asterisks (*) denote statistically significant differences compared to the vehicle control group (*p* < 0.05).

Table 2 summarizes the dose–effect parameters, including the median-effect dose (*Dm*), slope (*m*), and correlation coefficient (*r*), for BEA, ENN B, and MON, both individually and in combination. Among the individual mycotoxins, BEA showed the highest cytotoxic potency, with the lowest Dm value (1.69 μM), followed by ENN B (5.59 μM), while MON was the least potent, with a much higher Dm value (66.07 μM).

**TABLE 2:** Dose–effect parameters of BEA, ENN B, and MON in human Jurkat T cells following combined treatments.

Mycotoxins	Dose-effect parameters		
	*Dm* (µM)	*m*	*r*
BEA + ENN B	2.28692	0.56745	0.98331
BEA + MON	20.5313	0.47175	0.99340
ENN B + MON	27.3267	0.42638	0.98704
BEA + ENN B + MON	7.08757	0.67829	0.97361

*Dm* (median-effect dose) values for mixture treatments represent the total summed molar concentration (µM) of all constituent mycotoxins in the combination required to produce 50% cytotoxicity (Fa = 0.50). All parameters are presented as mean ± SD, from three independent biological experiments (n = 3).

For combination treatments, BEA + ENN B resulted in a lower Dm value (2.29 μM) compared to ENN B alone, indicating that BEA enhances the cytotoxic effect of ENN B. Similarly, the BEA + MON and ENN B+ MON combinations showed lower Dm values (20.53 μM and 27.33 μM, respectively) than MON alone, suggesting improved cytotoxicity when MON is combined with other toxins. The three-toxin combination (BEA + ENN B+ MON) had a Dm value of 7.09 μM, showing a clear increase in toxicity compared to MON alone, although it remained less potent than BEA alone. The slope (*m*) values indicate that most dose–response curves follow a typical sigmoidal pattern, and the high correlation coefficients (*r* > 0.94) confirm a good fit of the data to the median-effect model. Overall, the median-effect parameters (Dm) quantitatively reflect the shifts in overall mixture potency compared to individual compounds.

In line with these dose–response findings, the interactions between BEA, ENN B, and MON at low fractional-effect levels were evaluated evaluating interaction patterns at low to moderate fractional-effect levels (IC_10_ – IC_50_) using the combination index (CI)–fractional effect (fa) plots derived from the Chou–Talalay method (Figure 4). To accurately define interaction strengths and eliminate ambiguity near biological additivity, CI values were categorized using the graded Chou–Talalay terminology framework, where CI = 0.90–1.10 was defined as nearly additive to account for inherent experimental variability in cell-based assays. The results demonstrated that the nature of interactions varied depending on the toxin combinations and the level of effect (fa). For the BEA + ENN B combination (Figure 4A), strong synergism (CI = 0.199) was observed at IC_10_, which transitioned to synergism at IC_25_ (CI = 0.367) and IC_50_ (CI = 0.679). However, at higher effect levels, the interaction shifted toward moderate antagonism at IC_75_ (CI = 1.263) and antagonism at IC_90_ (CI = 2.360). In the BEA + MON combination (Figure 4B), very strong synergism (CI = 0.100) and strong synergism (CI = 0.282) were observed at lower effect levels (IC_10_ –IC_25_), which shifted to moderate synergism at IC_50_ (CI = 0.814). As the effect level further increased, the interaction transitioned into antagonism at IC_75_ (CI = 2.418) and strong antagonism at IC_90_ (CI = 7.381) Similarly, the ENN B+ MON combination (Figure 4C) exhibited very strong synergism at IC_10_ (CI = 0.051) and strong synergism at IC_25_ (CI = 0.208), progressing to slight synergism at IC_50_ (CI = 0.860), followed by a marked shift toward strong to very strong antagonism at higher effect levels (IC_75_ – IC_90_: CI = 3.585–15.101).

**FIGURE 4:**
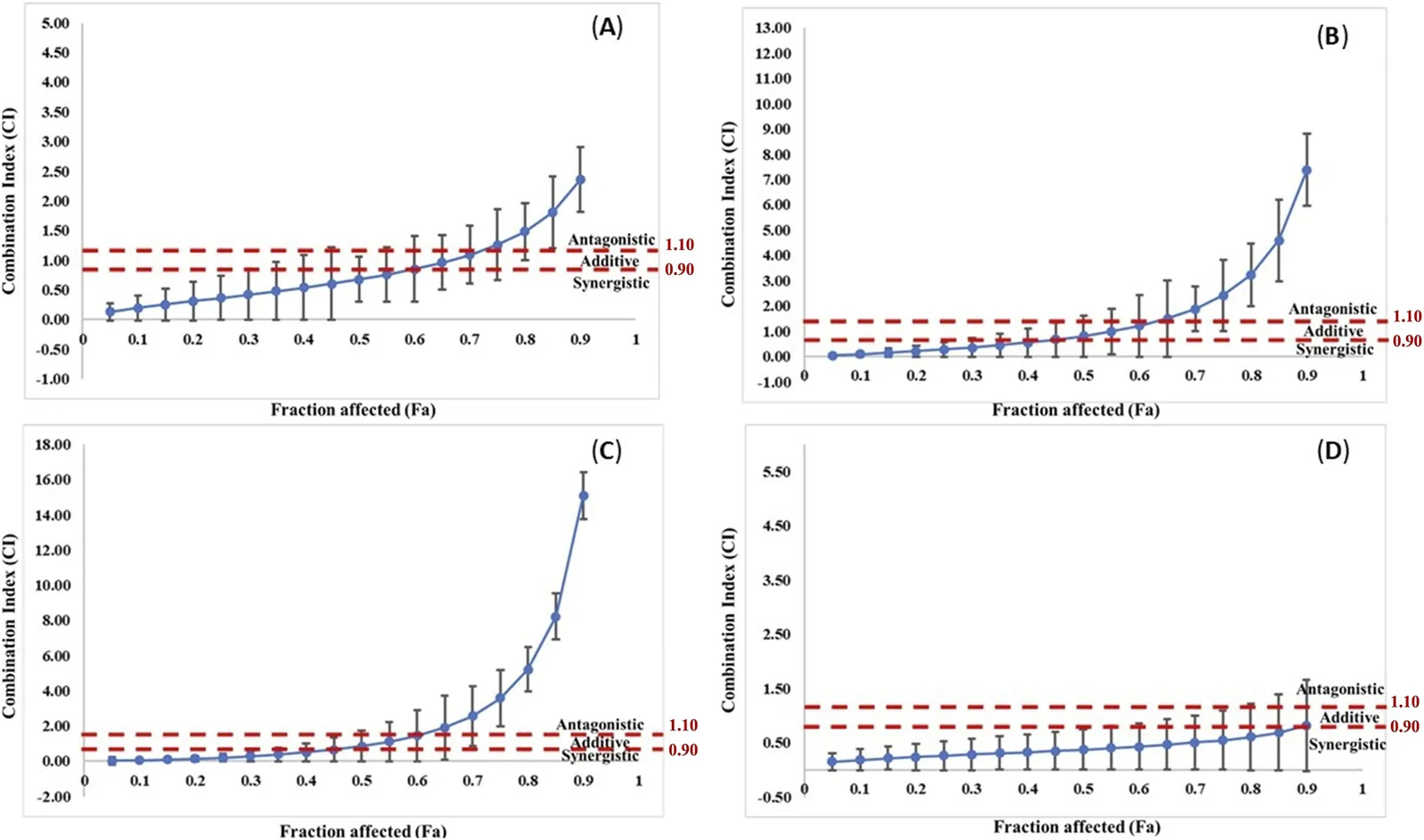
Combination index ( CI ) versus fraction affected ( Fa ) curves for beauvericin (BEA), enniatin B (ENN B), and moniliformin (MON) in human Jurkat T cells. CI – Fa plots were generated using the Chou–Talalay median-effect method for cells exposed to BEA + ENN B **(A)**, BEA + MON **(B)**, ENN B+ MON **(C)**, and the ternary mixture BEA + ENN B+ MON **(D)** for 24 h. Each data point represents the mean CI±SD derived from three independent biological experiments ( n=3 ) at specified fractional effect levels. The horizontal dashed line ( CI=1 ) denotes additivity ( 0.90≤CI≤1.10 ); values below 0.90 ( CI<0.90 ) represent synergism, whereas values above 1.10 ( CI>1.10 ) indicate antagonism.

In contrast, the ternary combination of BEA + ENN B+ MON (Figure 4D) demonstrated Combination Index (CI) values consistently below 1 across all evaluated fractional effect levels (fa = 0.1–0.9). Specifically, the ternary mixture displayed strong synergism at IC_10_ (CI = 0.185) and IC_25_ (CI = 0.262), synergism at IC_50_ (CI = 0.376) and IC_75_ (CI = 0.549), and moderate synergism at IC_90_ (CI = 0.818). This pattern confirms sustained synergistic interactions across the entire evaluated concentration range, without shifting toward antagonism even at higher fractional-effect levels. Overall, these findings indicate that combinations of BEA, ENN B, and MON produce synergistic cytotoxic effects predominantly at low to moderate levels of cell inhibition (IC_10_ – IC_50_), while higher effect levels in binary mixtures tend to shift the interactions toward antagonism. Importantly, defining CI = 0.90–1.10 as nearly additive ensured that minor experimental fluctuations around unity were not mischaracterized as genuine biological synergism or antagonism.

Table 3 presents the Combination Index (CI) and dose‐reduction index (DRI) values for BEA, ENN B, and MON in human Jurkat T cells at different effect levels (IC_10_–IC_90_), allowing evaluation of their interaction patterns. Overall, the CI analysis demonstrates synergistic interactions for combined treatments at low effect levels (IC_10_–IC_50_). At these levels, most combinations—including BEA + ENN B, BEA + MON, ENN B + MON, and the ternary mixture (BEA + ENN B + MON)— exhibited CI values below 0.90, representing distinct levels of synergism ranging from slight to very strong synergism. This means that the toxins enhance each other’s effects, resulting in greater cell inhibition than expected from individual treatments. In the same range, DRI values greater than 1 were observed, suggesting that lower doses of each toxin are required in combination to achieve the same level of cytotoxicity. This reflects a potential toxicological concern, as lower concentrations of each individual toxin in combination are sufficient to exert equivalent cytotoxic responses.

**TABLE 3:** Combination index (CI) and dose‐reduction index (DRI) values for cytotoxic interactions of BEA, ENN B, and MON in human Jurkat T cells.

Mycotoxins	IC10			IC25			IC50			IC75			IC90		
	CI	DRI	​	CI	DRI	​	CI	DRI	​	CI	DRI	​	CI	DRI	​
BEA + ENN B	0.19892	7.90294 13.8146	SS	0.36666	4.51742 6.88262	S	0.67889	2.58222 3.42902	S	1.26284	-	MA	2.36012	-	A
BEA + MON	0.09963	12.987 44.1815	VSS	0.28156	5.01246 12.1862	SS	0.81441	1.93461 3.36118	MS	2.41791	-	A	7.38064	-	SA
ENN B+ MON	0.05084	29.7743 57.9463	VSS	0.20808	7.81768 12.4747	SS	0.85954	2.05265 2.68557	SLS	3.5851	-	SA	15.101	-	SA
BEA + ENN B+ MON	0.18519	10.076 17.6132 34.2785	SS	0.26163	7.90296 12.0407 19.2135	SS	0.37567	6.19856 8.23128 10.7694	S	0.54906	4.86175 5.62707 6.03634	S	0.81776	3.81324 3.84678 3.38344	MS

CI, values were classified according to the graded Chou–Talalay terminology framework: CI < 0.10, very strong synergism (VSS); 0.10 ≤ CI < 0.30, strong synergism (SS); 0.30 ≤ CI < 0.70, synergism (S); 0.70 ≤ CI < 0.85, moderate synergism (MS); 0.85 ≤ CI < 0.90, slight synergism (SLS); 0.90 ≤ CI ≤ 1.10, nearly additive (Add); 1.10 < CI ≤ 1.20, slight antagonism (SLA); 1.20 < CI ≤ 1.45, moderate antagonism (MA); 1.45 < CI ≤ 3.30, antagonism (A); and CI > 3.30, strong to very strong antagonism (SA). Values within CI, 0.90–1.10 account for inherent experimental variability around pure additivity. DRI >1 indicates a favorable dose reduction, DRI, 1 indicates no dose reduction, and DRI <1 indicates that dose reduction is not supported.

However, at higher effect levels (IC_75_–IC_90_), the interaction patterns of some binary combinations (BEA + ENN B, BEA + MON, and ENN B + MON) shifted, yielding CI values greater than 1.10, indicating moderate to strong antagonistic effects. This suggests that at higher concentrations, these toxins may interfere with each other’s activity, reducing overall cytotoxic potency. In contrast, the ternary combination (BEA + ENN B + MON) consistently maintained CI values below 0.90 (ranging from strong to moderate synergism) and DRI >1 across all effect levels, indicating stable synergistic interactions and sustained toxicological hazards even at higher exposure levels.

To further elucidate the nature of these interactions, the polygonogram analysis at a fractional effect of 0.5 (Fa = 0.5), representing a moderate cytotoxic level (IC_50_), demonstrates the interaction patterns among BEA, ENN B, and MON in mixture treatments (Figure 5). All connecting lines between the compounds are shown as thick solid lines, indicating synergistic interactions for each binary combination (BEA + ENN B, BEA + MON, and ENN B + MON), as well as for the ternary mixture (BEA + ENN B + MON). These findings are fully consistent with the graded CI interaction analysis at IC_50_, where combinations exhibited synergism to slight synergism (CI = 0.376–0.860) and DRI values above 1. Together, these results confirm that, at low to moderate fractional-effect levels, the three mycotoxins interact in a cooperative manner, enhancing overall cytotoxicity beyond expected additive effects. The agreement between the polygonogram and CI-based analyses strengthens the conclusion that synergism is predominant under sub-maximal cytotoxic conditions, highlighting the potential risk of co-exposure to BEA, ENN B, and MON mixtures.

**FIGURE 5:**
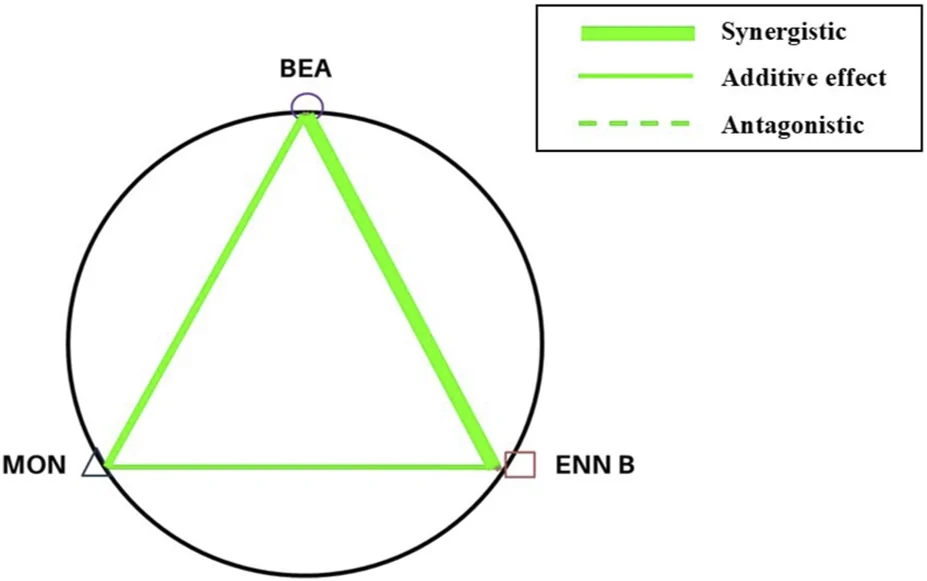
Polygonogram illustrating the interaction patterns among BEA, ENN B, and MON at a fractional effect of 0.5 (fa = 0.5) in human Jurkat T cells. Thick solid lines indicate synergistic interactions between toxin pairs, while the triangular connection represents synergism in the ternary combination. The results demonstrate consistent synergistic effects among all combinations at this effect level, supporting synergistic interactions under sub-maximal effect conditions based on Combination Index analysis.

### 3.3 Impact of individual and combined exposure to BEA, ENN B, and MON on cellular oxidative stress

To assess the role of oxidative stress in mycotoxin-induced cytotoxicity, intracellular ROS generation was evaluated in human Jurkat T cells following exposure to BEA, ENN B, and MON for 24 h. Exposure to BEA, ENN B, and MON resulted in a concentration-dependent increase in intracellular ROS levels relative to the vehicle control group (*p* < 0.05). Among the individual mycotoxins, BEA-treated cells showed higher ROS signal intensity, followed by ENN B, while MON exhibited a weaker response. This concentration-dependent elevation in ROS levels closely paralleled the reduction in cell viability (Figure 6). Combined exposure to BEA, ENN B, and MON resulted in a marked increase in intracellular ROS generation in human Jurkat T cells. All binary combinations (BEA + ENN B, BEA + MON, and ENN B+ MON) exhibited a clear concentration-dependent increasing trend in ROS levels relative to the vehicle control (*p* < 0.05). Among the binary mixtures, combinations containing BEA (BEA + ENN B and BEA + MON) exhibited higher relative ROS signals than the ENN B+ MON combination. The ternary combination (BEA + ENN B+ MON) showed the highest descriptive ROS levels across the tested concentration range. Overall, these results reflect descriptive trends showing elevated ROS-associated fluorescence signals in combination treatments, which parallel the concentration-dependent decreases in cell viability. As formal statistical interaction modeling was not performed for ROS endpoints, these trends are presented purely as descriptive observations rather than statistically established mixture-versus-single-agent interaction effects. (Figure 7).

**FIGURE 6:**
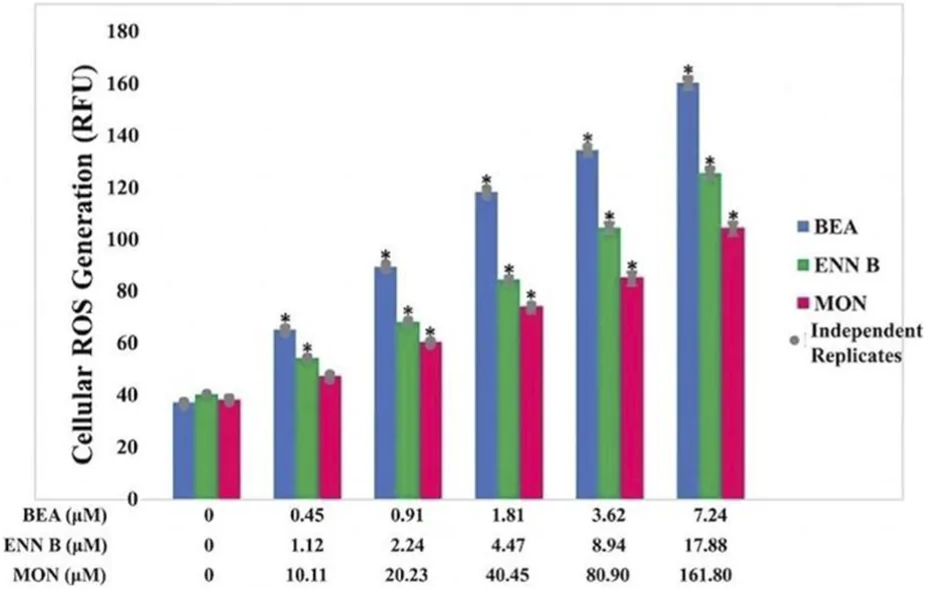
Effects of BEA, ENN B, and MON on intracellular reactive oxygen species (ROS) generation in human Jurkat T cells. Cells were exposed to increasing concentrations of BEA (0–7.24 μM), ENN B (0–17.88 μM), and MON (0–161.80 μM) for 24 h. ROS levels were measured using a Total ROS assay and expressed as relative fluorescence units (RFU). Data represent Mean ± SD of three independent biological experiments (n = 3), with individual experimental values shown as overlayed data points. Asterisks (*) indicate statistically significant differences compared to the control group (*p* < 0.05).

**FIGURE 7:**
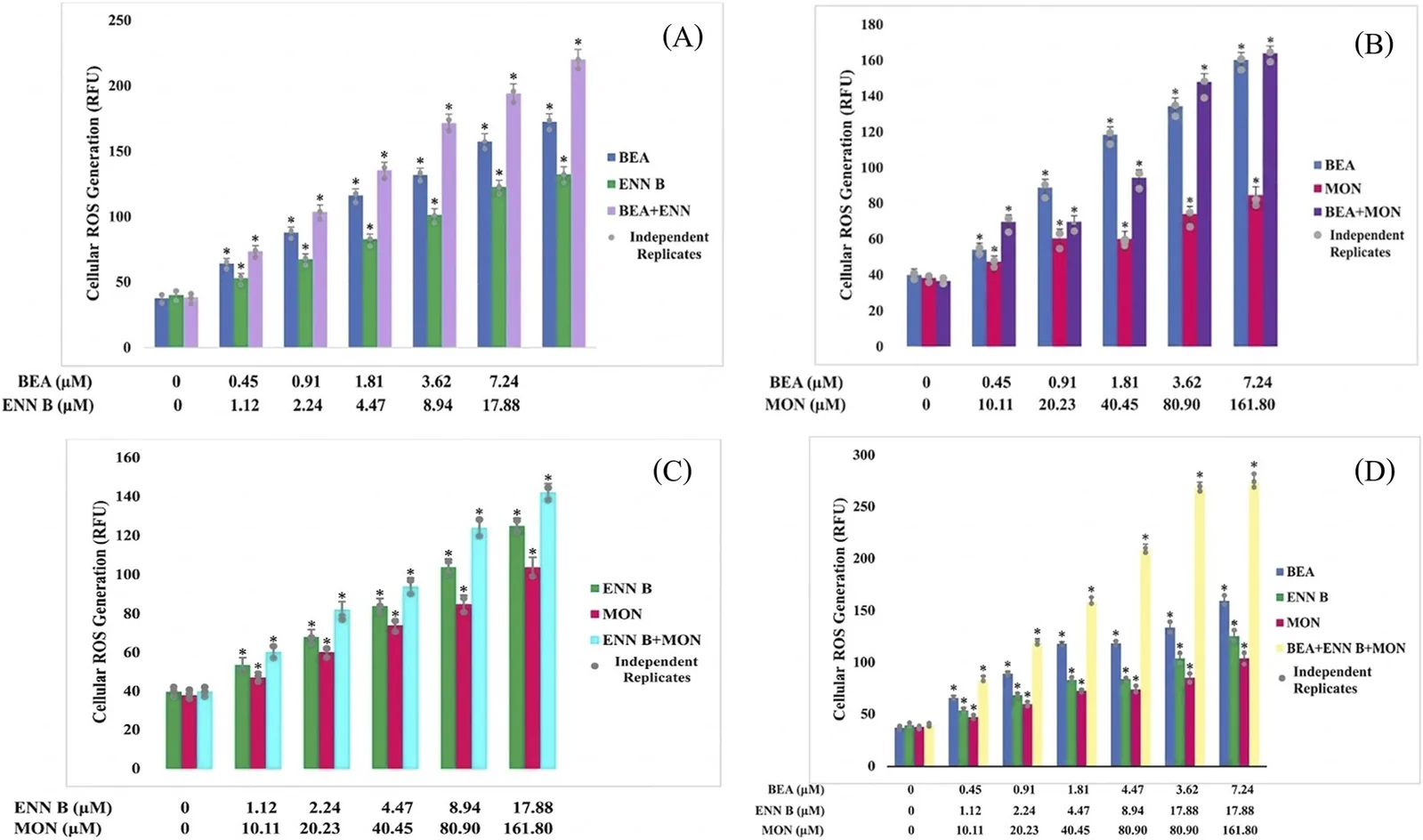
Effects of BEA, ENN B, and MON, individually and in combination, on intracellular reactive oxygen species (ROS) generation in human Jurkat T cells. Cells were exposed to increasing concentrations of BEA, ENN B, and MON, alone or in binary and ternary combinations, for 24 h. ROS levels were measured using a Total ROS assay and expressed as relative fluorescence units (RFU) **(A)** BEA + ENN B **(B)** BEA + MON **(C)** ENN B+ MON, and **(D)** BEA + ENN B+ MON. Data represent Mean ± SD of three independent biological experiments (n = 3), with individual experimental values shown as overlayed data points. Asterisks (*) indicate statistically significant differences compared to the control group (*p* < 0.05).

## 4 Discussion

The present study provides experimental evidence showing that the emerging *Fusarium* mycotoxins beauvericin (BEA), enniatin B (ENN B), and moniliformin (MON) exert measurable cytotoxic effects in human Jurkat T lymphocytes. In addition, the results demonstrate that combined exposure to these toxins can produce interaction patterns that differ from those observed for individual compounds. Among the tested mycotoxins, BEA showed the highest cytotoxic potency, followed by ENN B and MON, with IC_50_ values of 1.81, 4.47, and 40.45 µM, respectively. This toxicity ranking agrees with previous toxicological studies indicating that cyclic hexadepsipeptides such as BEA and ENN generally display stronger cytotoxic effects than MON in mammalian cells (Leslie, 2008).

The higher toxicity of BEA and ENN B can largely be explained by their chemical structure and biological activity. Both compounds belong to the family of cyclic depsipeptides and possess ionophoric properties (Yoshinari et al., 2025; Aufy et al., 2023). This allows them to form complexes with cations and transport ions across biological membranes. As a consequence, these toxins disrupt cellular ion balance, particularly by increasing intracellular calcium levels (Gielecińska et al., 2024; Lai et al., 2024). Elevated Ca^2+^ levels can disturb mitochondrial membrane potential and activate several apoptotic signaling pathways, eventually leading to loss of cell viability (Jestoi, 2008; Prosperini et al., 2013). Increased intracellular calcium may also stimulate the production of reactive oxygen species (ROS), which further enhances cellular stress (De Nicolo et al., 2023; Görlach et al., 2015). ENN B shares similar ionophoric properties because of its structural similarity to BEA, although its cytotoxic potency is often slightly lower depending on the cell type and experimental conditions (Bertero et al., 2020; Ivanova et al., 2006). Differences in lipophilicity, membrane permeability, and cellular uptake may explain the variations in toxicity observed between BEA and ENN B.

In contrast, MON is structurally very different from BEA and ENNs and therefore exhibits a different mechanism of toxic action. MON is a small and highly polar molecule that mainly interferes with cellular energy metabolism (Kamyar et al., 2004). Previous studies have shown that MON can inhibit several important metabolic enzymes, including pyruvate dehydrogenase and enzymes involved in oxidative phosphorylation, which ultimately disrupts ATP production and mitochondrial function (Uhlig et al., 2004). Because of this mechanism, MON toxicity is often associated with metabolic disturbances and cardiotoxic effects rather than strong acute cytotoxicity in cultured cells. This difference in mode of action may explain the higher IC_50_ value observed for MON in the present study compared with BEA and ENN B. Similar observations have been reported in other toxicological studies. For example, Kamyar et al. (2007) reported that MON generally shows lower acute cytotoxicity *in vitro* compared with cyclic depsipeptide mycotoxins, although metabolic effects may still occur at lower concentrations.

Further mechanistic evidence has shown that the ionophoric activity of BEA and ENNs allows them to transport mono- and divalent cations across lipid bilayers. This process disrupts intracellular ionic balance and triggers several cellular stress responses (Jiang et al., 2024). Tonshin et al. (2010) also demonstrated that BEA and ENNs can damage cellular membranes and mitochondrial structures, which leads to impaired energy production and eventually cell death. These findings help explain the strong cytotoxic effects observed in the present study. Importantly, previous studies focusing on immune-related cell models have also highlighted the high sensitivity of lymphoid cells to cyclic depsipeptide mycotoxins. For example, Dornetshuber et al. (2007) showed that BEA exposure led to mitochondrial membrane potential collapse, cytochrome c release, and activation of caspase-3 and caspase-9, which are key regulators of the intrinsic apoptosis pathway. Similar apoptotic responses have been reported in other immune and hematopoietic cell models exposed to BEA and ENNs (Bikomeye et al., 2022). Because T lymphocytes are particularly sensitive to mitochondrial stress and oxidative imbalance, disruption of mitochondrial function can rapidly reduce cell viability and alter immune signaling pathways (Xia et al., 2022). Therefore, the results of the present study further support the idea that BEA and ENN B have pronounced cytotoxic activity in human immune cells. Considering the important role of immune cells in host defense, such effects may have implications for understanding the potential immunotoxic risks associated with dietary exposure to emerging Fusarium mycotoxins. In addition to the reduced cell viability, another important observation in this study was the concentration-dependent elevation in intracellular ROS signals following exposure to BEA, ENN B, and MON (Giri et al., 2023). The generation of oxidative stress has been widely suggested in the literature as a key mechanism involved in the toxicity of many Fusarium-derived metabolites. Previous studies have demonstrated that BEA and ENNs can disrupt mitochondrial function, which leads to excessive ROS production and oxidative damage to cellular macromolecules (Prosperini et al., 2013). In literature, increased ROS levels are reported to trigger lipid peroxidation, DNA damage, and activation of apoptosis signaling pathways, which ultimately contribute to reduced cell survival. Oxidative stress may be particularly important in immune cells. In T lymphocytes, excessive ROS production can interfere with immune signaling pathways and affect cytokine production (Ekwomadu et al., 2021). As a result, prolonged oxidative stress may weaken immune responses during chronic exposure to mycotoxins. Therefore, the increase in ROS signals observed in the present study closely parallels the marked reduction in cell viability, supporting a potential association between elevated ROS levels and mycotoxin exposure, although formal interaction modeling and direct mechanistic causality were not established.

Beyond the toxicity of individual compounds, an important aspect of this study was the evaluation of mixture interactions among BEA, ENN B, and MON using the Chou–Talalay method (Bloch et al., 2023). Based on the graded Chou–Talalay framework, combination index (CI) analysis demonstrated distinct, concentration-dependent shifts in interaction patterns across fractional-effect levels (IC_10_ – IC_90_). At lower effect levels (IC_10_ – IC_50_), all binary combinations exhibited interactions ranging from very strong to slight synergism (CI = 0.051–0.860), whereas higher inhibitory levels (IC_75_ – IC_90_) shifted toward moderate to strong antagonism (CI = 1.263–15.101). In contrast, the ternary mixture maintained sustained synergism across the entire effect range (CI = 0.185–0.818). At lower fractional-effect levels, the combined disruption of ion balance, mitochondrial function, and metabolic pathways may amplify cellular stress responses, resulting in synergistic toxicity (Machiela et al., 2020). Defining CI = 0.90–1.10 as a nearly additive zone further ensured that subtle experimental variations near theoretical additivity were appropriately distinguished from genuine biological synergism or antagonism.

At higher concentrations, however, the interaction pattern shifted toward antagonism in all binary combinations. This concentration-dependent transition may reflect several complementary mechanisms rather than a simple saturation effect. Because BEA and ENN B are ionophoric compounds that disrupt transmembrane cation homeostasis, excessive ion influx at high concentrations may activate adaptive cellular stress responses, including antioxidant defense systems and mitochondrial quality-control pathways, thereby partially limiting further toxicity (Søderstrøm et al., 2022). In addition, excessive ROS accumulation may trigger compensatory redox-regulatory mechanisms that reduce the incremental cytotoxic contribution of each toxin. Competition between structurally related ionophores for membrane incorporation, ion transport, or shared intracellular targets may also diminish their combined efficacy at higher concentrations, resulting in an apparent antagonistic interaction (Chen et al., 2025).

In contrast, the ternary combination maintained synergistic interactions across all evaluated effect levels (IC_10_–IC_90_). One possible explanation is that the coexistence of BEA, ENN B, and MON simultaneously perturbs complementary cellular processes. While BEA and ENN B primarily disrupt membrane ion homeostasis and mitochondrial function through their ionophoric activity, MON predominantly interferes with cellular energy metabolism (Tonshin et al., 2010). The convergence of these distinct upstream mechanisms on common downstream events—including mitochondrial dysfunction, oxidative stress, ATP depletion, and apoptotic signaling—may overwhelm compensatory cellular responses and sustain synergistic cytotoxicity even at high effect levels. The descriptive elevation in ROS-associated fluorescence signals parallels the cytotoxic responses observed across treatment groups. However, because statistical interaction was not assessed for ROS and ROS signals were not formally compared between mixtures and single agents, elevated ROS signals cannot be inferred as statistically enhanced by combination treatments or as the mechanistic driver of the modeled cytotoxic synergy. However, because specific downstream endpoints such as mitochondrial membrane depolarization, ATP depletion, and apoptotic execution markers were not directly evaluated in this study, the hypothesis that oxidative stress acts as a key mediator remains a biologically plausible interpretation derived from literature that warrants further experimental validation. Nevertheless, additional mechanistic studies evaluating mitochondrial membrane potential, intracellular ATP levels, calcium homeostasis, and apoptosis-related signaling pathways are required to experimentally validate these proposed mechanisms (Kong et al., 2025).

The interpretation of these interaction patterns should also be considered in light of the methodological approach used. In the present study, mixture interactions were evaluated using the Chou–Talalay method, which is based on the median-effect principle and enables quantitative determination of synergistic, additive, or antagonistic effects through the combination index (CI). A key advantage of this approach is its use of fixed-ratio designs normalized to compound potency, allowing standardized comparison across a wide range of effect levels (Chou, 2010). In contrast, other models such as Bliss independence and Loewe additivity are also commonly applied in mixture toxicology. The Bliss model assumes independent modes of action and is often suitable for compounds acting through different pathways, whereas the Loewe model is based on the concept of dose equivalence and is more appropriate for compounds with similar mechanisms of action (Foucquier and Guedj, 2015). Compared to these approaches, the Chou–Talalay method provides a more comprehensive evaluation across multiple effect levels (fa), which is particularly advantageous for identifying shifts between synergistic and antagonistic interactions. However, it should be noted that this method relies on assumptions that may not fully capture the complexity of interactions between mechanistically distinct compounds such as BEA, ENN B, and MON. Therefore, while the CI-based analysis offers valuable quantitative insight, complementary approaches may be required to fully elucidate the biological interactions underlying mixture toxicity.

Interestingly, the ternary combination of BEA, ENN B, and MON in the present study showed predominantly synergistic interactions across the entire evaluated effect range (IC_10_–IC_90_). This finding is particularly important from a food safety perspective. In real dietary exposure scenarios, humans are rarely exposed to a single mycotoxin. Instead, they are often exposed to complex mixtures of fungal metabolites present in contaminated cereals and cereal-based products (Goessens et al., 2024). Surveys of grain commodities worldwide have repeatedly reported the co-occurrence of multiple Fusarium mycotoxins, including BEA and enniatins, often together with other regulated toxins such as deoxynivalenol and fumonisins (Jestoi et al., 2004; Uhlig et al., 2013). The predominance of synergistic effects observed in the ternary mixture therefore suggests that combined exposure may pose a higher toxicological risk than predicted from single-toxin data alone.

Beyond their frequent occurrence, emerging Fusarium mycotoxins raise additional concern because they often fall outside routine monitoring programs and established maximum limits (Ekwomadu et al., 2021). This regulatory gap largely reflects the still-limited toxicological database available for these compounds, particularly under co-exposure scenarios. As analytical methods continue to improve, the detection frequency of BEA, ENN B, and MON in food matrices is expected to increase, potentially widening the gap between exposure evidence and risk characterization (Ahuja et al., 2023; Devos et al., 2022). Consequently, these metabolites may represent an underrecognized component of the total mycotoxin burden in the food supply. It should be noted, however, that the concentration ranges evaluated in the present study are higher than the plasma or tissue concentrations currently reported following typical dietary exposure to BEA, ENN B, and MON, which are generally within the nanomolar to sub-micromolar range. Therefore, the present findings should not be interpreted as directly representing realistic human exposure scenarios. Instead, the selected concentration range was intended to establish complete dose–response relationships and to enable quantitative assessment of mixture interactions across a broad spectrum of effect levels using the Chou–Talalay method. Such an approach is widely employed in mechanistic *in vitro* toxicology to identify potential interaction patterns, including synergism and antagonism, under controlled experimental conditions. Nevertheless, extrapolation of these findings to human health risk should be made with caution, as *in vitro* systems do not fully account for toxicokinetic processes such as absorption, distribution, metabolism, and excretion. Future studies employing chronic low-dose exposure models, physiologically relevant concentrations, and more complex biological systems will be essential for improving the translational relevance of these findings and refining mixture-based risk assessment of emerging Fusarium mycotoxins. Mechanistically, the physicochemical diversity among BEA, ENN B, and MON suggests the potential for non-linear and pathway-convergent interactions at the cellular level (Khan et al., 2024). The lipophilic ionophoric nature of BEA and ENN B enables direct perturbation of membrane integrity and ionic gradients, whereas MON primarily interferes with metabolic processes linked to cellular energy production (Aufy et al., 2023; Tonshin et al., 2010). When present simultaneously, these mechanistically distinct toxins may converge on common downstream events such as mitochondrial impairment, oxidative stress, and programmed cell death (Korotkov, 2023). The use of Jurkat T cells as a model system in this study also provides useful insight into the potential immunotoxic effects of emerging mycotoxins. T lymphocytes play a central role in adaptive immune responses, and impairment of these cells could influence host defense mechanisms (Singh et al., 2022; Carrasco Padilla et al., 2022). Previous studies have shown that several mycotoxins, including trichothecenes and fumonisins, can affect immune cell viability and function (Pestka, 2010). The sensitivity of Jurkat T cells observed in the present study suggests that emerging mycotoxins such as BEA and ENN B may also affect immune-related processes. Although Jurkat T cells provide a well-established and reproducible model for investigating T-cell responses and immunotoxic mechanisms, they are derived from an immortalized leukemia cell line and therefore may not fully recapitulate the physiological characteristics of normal human immune cells. Consequently, validation of the present findings in primary human immune cells, such as peripheral blood mononuclear cells (PBMCs), would further strengthen the translational relevance of these observations and should be considered in future studies.

Despite these findings, several limitations should be considered. A key limitation of the present study is the reliance on a single immortalized cell line under acute 24 h exposure conditions. Although human Jurkat T cells serve as a widely accepted *in vitro* model for initial cytotoxicity screening, as transformed leukemic cells they exhibit altered metabolism and stress response thresholds compared to primary human lymphocytes. Consequently, these findings cannot fully represent the complexity of primary immune cells or whole-organism exposures, and potential implications for human immunotoxicity remain biologically plausible hypotheses that require confirmation in primary cell models and chronic exposure designs. In addition, while intracellular ROS generation was evaluated as a complementary indicator to support the cytotoxicity results, detailed interaction-based statistical modeling (e.g., two-way ANOVA) and quantitative ROS dose–response modeling was not conducted. Consequently, evaluating ROS interactions at a direct quantitative level was beyond the scope of this study. Additionally, a methodological limitation includes the absence of a conventional chemical positive cytotoxicity control during cell viability and ROS assays. Although assay performance, signal reproducibility, and plate-to-plate consistency were rigorously maintained through strict normalization relative to the vehicle control group (vehicle variability <5%), multiple independent biological replicates (n ≥ 3), and high linear correlation coefficients (*r* > 0.95) in median-effect plots, incorporating standard positive cytotoxicity controls will further bolster assay validation in future studies. Furthermore, intracellular ROS fluorescence was expressed as background-corrected RFU values relative to controls without direct normalization to the remaining viable cell number per well. Consequently, the absence of cell-number or viability normalization limits direct quantitative comparison across treatment conditions with markedly different cell viability levels, as multiple cellular factors—including probe loading, esterase activity, membrane integrity, and fluorescence retention—may complexly influence the measured signal. Future studies incorporating cell-number or dual-staining normalization will further refine these single-cell oxidative dynamics. Furthermore, downstream mechanistic events such as mitochondrial membrane potential (ΔΨm) collapse, apoptosis signaling pathways, and gene expression changes were not investigated in the present study. Since BEA and ENN B are known ionophores capable of disrupting mitochondrial function, assessment of these endpoints would provide direct mechanistic evidence supporting the proposed mode of action and should be addressed in future investigations (Egea et al., 2020). Overall, the results of this study contribute to the growing evidence that emerging Fusarium mycotoxins can exert significant biological effects in human cells. The observation of synergistic interactions, particularly in ternary mixtures, highlights the importance of considering mixture toxicity in mycotoxin risk assessment. As analytical techniques continue to reveal increasingly complex contamination patterns in food commodities, integrating mixture toxicology into food safety evaluations will become increasingly important for accurately assessing potential health risks.

## 5 Conclusion

This study demonstrates that BEA, ENN B, and MON exert significant cytotoxic effects on Jurkat T cells, both individually and in combination. Among the tested mycotoxins, BEA exhibited the highest potency, followed by ENN B, while MON showed comparatively lower toxicity. Importantly, combined exposure resulted in lower cell viability, particularly at low fractional-effect levels (IC_10_ – IC_50_), where interactions ranging from very strong to slight synergism (CI < 0.90) were consistently observed for binary combinations, while the ternary mixture maintained sustained synergism (CI = 0.185–0.818) across all evaluated effect levels (IC_10_ – IC_90_). Overall, these findings highlight that co-exposure to multiple Fusarium mycotoxins at sub-maximal inhibitory concentrations leads to enhanced reductions in cell viability relative to vehicle control, characterized by synergistic interactions (as defined by the Combination Index) and elevated ROS-associated fluorescence signals in Jurkat T cells. This underscores the importance of considering mixture-based approaches in food safety risk assessment rather than evaluating individual contaminants alone.

## Data Availability

The original contributions presented in the study are included in the article/Supplementary Material, further inquiries can be directed to the corresponding author.

## References

[B1] Aghzaf S. Poli J.-P. Brunel M. Costa J. Lorenzi V. Guinoiseau E. (2025). Characterization of the bacterial and fungal diversity in habitats of Corsica Island. Appl. Environ. Microbiol. 91 (10), e0075625. 10.1128/aem.00756-25 PMC12542654 41031848

[B2] Ahuja V. Singh A. Paul D. Dasgupta D. Urajová P. Ghosh S. (2023). Recent advances in the detection of food toxins using mass spectrometry. Chem. Res. Toxicol. 36 (12), 1834–1863. 10.1021/acs.chemrestox.3c00241 PMC10731662 38059476

[B3] Alassane-Kpembi I. Kolf-Clauw M. Gauthier T. Abrami R. Abiola F. A. Oswald I. P. (2013). New insights into mycotoxin mixtures: the toxicity of low doses of Type B trichothecenes on intestinal epithelial cells is synergistic. Toxicol. Appl. Pharmacol. 272 (1), 191–198. 10.1016/j.taap.2013.05.023 23735874

[B4] Alassane-Kpembi I. Schatzmayr G. Taranu I. Marin D. Puel O. Oswald I. P. (2017). Mycotoxins co-contamination: methodological aspects and biological relevance of combined toxicity studies. Crit. Reviews Food Science Nutrition 57 (16), 3489–3507. 10.1080/10408398.2016.1140632 26918653

[B5] Ashton J. (2015). Drug combination studies and their synergy quantification using the Chou–Talalay method—letter. Cancer Res. 75, 2400. 10.1158/0008-5472.CAN-14-3763 25977339

[B6] Aufy M. Abdelaziz R. F. Hussein A. M. Topcagic N. Shamroukh H. Abdel-Maksoud M. A. (2023). Impact of enniatin B and beauvericin on lysosomal cathepsin B secretion and apoptosis induction. Int. J. Mol. Sci. 24 (3), 2030. 10.3390/ijms24032030 PMC9916760 36768354

[B7] Ball N. Bars R. Botham P. A. Cuciureanu A. Cronin M. T. D. Doe J. E. (2022). A framework for chemical safety assessment incorporating new approach methodologies within REACH. Archives Toxicol. 96 (3), 743–766. 10.1007/s00204-021-03215-9 PMC8850243 35103819

[B8] Behr A. C. Fæste C. K. Azqueta A. Tavares A. M. Spyropoulou A. Solhaug A. (2025). Hazard characterization of the mycotoxins enniatins and beauvericin to identify data gaps and improve risk assessment for human health. Archives toxicology 99 (5), 1791–1841. 10.1007/s00204-025-03988-3 PMC12085332 40137953

[B9] Benkerroum N. (2020). Chronic and acute toxicities of aflatoxins: mechanisms of action. Int. J. Environmen. Res. Public Health 17 (2), 423. 10.3390/ijerph17020423 31936320 PMC7013914

[B10] Bertero A. Fossati P. Tedesco D. E. A. Caloni F. (2020). Beauvericin and enniatins: *in vitro* intestinal effects. Toxins 12 (11), 686. 10.3390/toxins12110686 33138307 PMC7693699

[B11] Bikomeye J. C. Terwoord J. D. Santos J. H. Beyer A. M. (2022). Emerging mitochondrial signaling mechanisms in cardio-oncology: beyond oxidative stress. Am. J. Physiol. Heart Circ. Physiol. 323 (4), H702–H720. 10.1152/ajpheart.00231.2022 PMC9529263 35930448

[B12] Bloch D. Diel P. Epe B. Hellwig M. Lampen A. Mally A. (2023). Basic concepts of mixture toxicity and relevance for risk evaluation and regulation. Archives toxicol. 97 (11), 3005–3017. 10.1007/s00204-023-03565-6 PMC10504116 37615677

[B13] Bosch J. Némethová E. Tláskal V. Brabcová V. Baldrian P. (2023). Bacterial, but not fungal, communities show spatial heterogeneity in European beech (Fagus sylvatica L.) deadwood. FEMS Microbiology Ecology 99 (4), fiad023. 10.1093/femsec/fiad023 PMC10065134 36906283

[B14] Bugingo C. Infantino A. Okello P. Perez-Hernandez O. Petrović K. Turatsinze A. N. (2025). From morphology to multi-omics: a new age of fusarium research. Pathog. (Basel, Switzerland) 14 (8), 762. 10.3390/pathogens14080762 PMC12389138 40872272

[B15] Carrasco Padilla C. Aguilar-Sopeña O. Gómez-Morón Á. Alegre Gómez S. Sánchez-Madrid F. Martin N. (2022). T Cell Activ. Effector Funct. Hum. Jurkat T Cell Model 178, 25–41. 10.1016/bs.mcb.2022.09.012 37516527

[B16] Chałaśkiewicz K. Kępka-Borkowska K. Starzyński R. R. Ogłuszka M. Borkowski M. Poławska E. (2025). Impact of aflatoxins on the digestive, immune, and nervous systems: the role of microbiota and probiotics in toxicity protection. Int. J. Mol. Sci. 26 (17), 8258. 10.3390/ijms26178258 PMC12428308 40943180

[B17] Chen Y. S. Tian H. X. Rong D. C. Wang L. Chen S. Zeng J. (2025). ROS homeostasis in cell fate, pathophysiology, and therapeutic interventions. Mol. Biomedicine 6 (1), 89. 10.1186/s43556-025-00338-8 PMC12572517 41162811

[B18] Chilenga C. Kasapila W. Masamba K. Manani T. Munkhuwa V. Ndhlovu B. (2025). Knowledge structure and evolution of masked mycotoxin research in Sub-Saharan Africa: a systematic review and network analysis approach. Toxicon X 28, 100232. 10.1016/j.toxcx.2025.100232 PMC12508825 41081273

[B81] Chou T. C. (2006). Theoretical basis, experimental design, and computerized simulation of synergism and antagonism in drug combination studies. Pharmacol. Rev. 58 (3), 621–681. 10.1124/pr.58.3.10 16968952

[B19] Chou T. C. (2010). Drug combination studies and their synergy quantification using the Chou-Talalay method. Cancer research 70 (2), 440–446. 10.1158/0008-5472.CAN-09-1947 20068163

[B20] Coulet F. Hymery N. Coton E. Coton M. (2025). Enniatin mycotoxins in food: a systematic review of global occurrence, biosynthesis, and toxicological impacts on *in vitro* human cell models. Compr. Rev. Food Sci. Food Saf. 24 (5), e70270. 10.1111/1541-4337.70270 PMC12433590 40946203

[B21] De Nicolo B. Cataldi-Stagetti E. Diquigiovanni C. Bonora E. (2023). Calcium and reactive oxygen species signaling interplays in cardiac physiology and pathologies. Antioxidants 12 (2), 353. 10.3390/antiox12020353 PMC9952851 36829912

[B22] Devos Y. Bray E. Bronzwaer S. Gallani B. Url B. (2022). Advancing food safety: strategic recommendations from the ‘ONE – Health, environment and society – conference 2022. EFSA J. 20 (11), e201101. 10.2903/j.efsa.2022.e201101 PMC9650440 36381115

[B23] Di Salvo E. Bartolomeo G. Vadalà R. Costa R. Cicero N. (2025). Mycotoxins in ready-to-eat foods: regulatory challenges and modern detection methods. Toxics 13 (6), 485. 10.3390/toxics13060485 PMC12197077 40559958

[B24] Dornetshuber R. Heffeter P. Kamyar M.-R. Peterbauer T. Berger W. Lemmens-Gruber R. (2007). Enniatin exerts p53-Dependent cytostatic and p53-Independent cytotoxic activities against human cancer cells. Chem. Res. Toxicol. 20 (3), 465–473. 10.1021/tx600259t 17326668

[B25] D’Amore T. Smaoui S. Varzakas T. (2025). Chemical food safety in Europe under the spotlight: principles, regulatory framework and roadmap for future directions. Foods 14 (9), 1628. 10.3390/foods14091628 PMC12071582 40361710

[B26] Egea G. Jiménez-Altayó F. Campuzano V. (2020). Reactive oxygen species and oxidative stress in the pathogenesis and progression of genetic diseases of the connective tissue. Antioxidants 9 (10), 1013. 10.3390/antiox9101013 33086603 PMC7603119

[B27] Ekwomadu T. I. Akinola S. A. Mwanza M. (2021). *Fusarium* mycotoxins, their metabolites (free, emerging, and masked), food safety concerns, and health impacts. Int. Journal Environ. Res. Public Health 18 (22), 11741. 10.3390/ijerph182211741 PMC8618243 34831498

[B28] Fărcaş A. C. (2024). “Food safety in cereal grains: contaminants, legislation, and mitigation strategies”. in Worldwide Megatrends in Food Safety and Food Security. Editors Marc R. A. Mureşan C. C. Narcisa Postolache A. (London: IntechOpen). 10.5772/intechopen.1007523

[B29] Foucquier J. Guedj M. (2015). Analysis of drug combinations: current methodological landscape. Pharmacol. Res. and Perspect. 3 (3), e00149. 10.1002/prp2.149 26171228 PMC4492765

[B30] Fremy J. Alassane-Kpembi I. Oswald I. P. Cottrill B. Van Egmond H. P. (2019). A review on combined effects of moniliformin and co-occurringFusarium toxins in farm animals. World Mycotoxin J. 12 (3), 281–292. 10.3920/WMJ2018.2405

[B31] Garofalo M. Payros D. Penary M. Oswald E. Nougayrède J. P. Oswald I. P. (2023). A novel toxic effect of foodborne trichothecenes: the exacerbation of genotoxicity. Environ. Pollut. (Barking, Essex 1987) 317, 120625. 10.1016/j.envpol.2022.120625 36410598

[B32] Gielecińska A. Kciuk M. Kontek R. (2024). The impact of calcium overload on cellular processes: exploring calcicoptosis and its therapeutic potential in cancer. Int. J. Mol. Sci. 25 (24), 13727. 10.3390/ijms252413727 PMC11679949 39769488

[B33] Giri P. Camarillo I. G. Sundararajan R. (2023). Enhancement of reactive oxygen species production in triple negative breast cancer cells treated with electric pulses and resveratrol. Explor. Target. Anti-tumor Ther. 4 (1), 42–56. 10.37349/etat.2023.00122 PMC10017187 36937321

[B34] Goessens T. Mouchtaris-Michailidis T. Tesfamariam K. Truong N. N. Vertriest F. Bader Y. (2024). Dietary mycotoxin exposure and human health risks: a protocol for a systematic review. Environ. Int. 184, 108456. 10.1016/j.envint.2024.108456 PMC10895515 38277998

[B35] Görlach A. Bertram K. Hudecova S. Krizanova O. (2015). Calcium and ROS: a mutual interplay. Redox Biol. 6, 260–271. 10.1016/j.redox.2015.08.010 26296072 PMC4556774

[B36] Gruber-Dorninger C. Novak B. Nagl V. Berthiller F. (2017). Emerging mycotoxins: beyond traditionally determined food contaminants. J. Agric. Food Chem. 65 (33), 7052–7070. 10.1021/acs.jafc.6b03413 27599910

[B37] Ivanova L. Skjerve E. Eriksen G. S. Uhlig S. (2006). Cytotoxicity of enniatins A, A1, B, B1, B2 and B3 from Fusarium avenaceum. Toxicon. 47(8), 868–876. 10.1016/j.toxicon.2006.02.012 16730043

[B38] Jestoi M. (2008). Emerging fusarium-mycotoxins fusaproliferin, beauvericin, enniatins, and moniliformin: a review. Crit. Rev. Food. Sci. Nutr. 48 (1), 21–49. 10.1080/10408390601062021 18274964

[B39] Jestoi M. Ritieni A. Rizzo A. (2004). Analysis of the fusarium mycotoxins fusaproliferin and trichothecenes in grains using gas chromatography−mass spectrometry. J. Agric. Food. Chem. 52 (6), 1464–1469. 10.1021/jf035130g 15030197

[B40] Jiang S. Lan Z. Zhang Y. Kang X. Zhao L. Wu X. (2024). Mechanisms by which exogenous substances enhance plant salt tolerance through the modulation of ion membrane transport and reactive oxygen species metabolism. Antioxidants 13 (9), 1050. 10.3390/antiox13091050 PMC11428486 39334709

[B41] Juan C. A. Pérez de la Lastra J. M. Plou F. J. Pérez-Lebeña E. (2021). The chemistry of reactive oxygen species (ROS) revisited: outlining their role in biological macromolecules (DNA, lipids and proteins) and induced pathologies. Int. Journal Mol. Sci. 22 (9), 4642. 10.3390/ijms22094642 PMC8125527 33924958

[B42] Kamyar M. Rawnduzi P. Studenik C. K K. Lemmens-Gruber R. (2004). Investigation of the electrophysiological properties of enniatins. Archives Biochem. Biophys. 429, 215–223. 10.1016/j.abb.2004.06.013 15313225

[B43] Kamyar R. K K. Rawnduzi P. Studenik C. Lemmens-Gruber R. (2007). Effects of moniliformin in presence of cyclohexadepsipeptides on isolated mammalian tissue and cells. Toxicol. In Vitro An International Journal Published Association BIBRA 20, 1284–1291. 10.1016/j.tiv.2006.03.001 16621435

[B44] Kanabus J. Bryła M. Leśnowolska-Wnuczek K. Waśkiewicz A. Twarużek M. (2026). Mycotoxins occurrence in herbs, spices, dietary supplements, and their exposure assessment. Toxins 18 (1), 20. 10.3390/toxins18010020 PMC12845980 41591166

[B45] Khan R. Anwar F. Ghazali F. M. (2024). A comprehensive review of mycotoxins: toxicology, detection, and effective mitigation approaches. Heliyon 10 (8), e28361. 10.1016/j.heliyon.2024.e28361 PMC11019184 38628751

[B46] Kolf-Clauw M. Sassahara M. Lucioli J. Rubira-Gerez J. Alassane-Kpembi I. Lyazhri F. (2013). The emerging mycotoxin, enniatin B1, down-modulates the gastrointestinal toxicity of T-2 toxin *in vitro* on intestinal epithelial cells and *ex vivo* on intestinal explants. Archives Toxicol. 87, 2233–2241. 10.1007/s00204-013-1067-8 23649843

[B47] Kong L. Dong J. Wāng Y. (2025). Mitochondrial homeostasis via Pink1 control alleviates nickel nanoparticle-induced reproductive damage in mouse spermatocyte GC-2 cells. Environ. Int. 206, 109910. 10.1016/j.envint.2025.109910 41248565

[B48] Korotkov S. M. (2023). Mitochondrial oxidative stress is the general reason for apoptosis induced by different-valence heavy metals in cells and mitochondria. Int. J. Mol. Sci. 24 (19), 14459. 10.3390/ijms241914459 PMC10572412 37833908

[B49] Kraft S. Buchenauer L. Polte T. (2021). Mold, mycotoxins and a dysregulated immune system: a combination of concern? Int. J. Mol. Sci. 22 (22), 12269. 10.3390/ijms222212269 PMC8619365 34830149

[B50] Lai Y. Gao F. f. Ge R. t. Liu R. Ma S. Liu X. (2024). Metal ions overloading and cell death. Cell Biol. Toxicol. 40 (1), 72. 10.1007/s10565-024-09910-4 PMC11335907 39162885

[B51] Leslie J. (2008). Mycotoxins: Detection Methods, Management, Public Health and Agricultural Trade. 10.1079/9781845930820.0000

[B52] Machiela E. Liontis T. Dues D. J. Rudich P. D. Traa A. Wyman L. (2020). Disruption of mitochondrial dynamics increases stress resistance through activation of multiple stress response pathways. FASEB J. Official Publication Fed. Am. Soc. Exp. Biol. 34 (6), 8475–8492. 10.1096/fj.201903235R 32385951 PMC7313680

[B53] Maphaisa T. C. Akinmoladun O. F. Adelusi O. A. Mwanza M. Fon F. Tangni E. (2025). Advances in mycotoxin detection techniques and the crucial role of reference material in ensuring food safety. A review. Food. Chem. Toxicol. 200, 115387. 10.1016/j.fct.2025.115387 40081789

[B54] Mischler S. André A. Chetschik I. Miescher Schwenninger S. (2024). Potential for the bio-detoxification of the mycotoxins enniatin B and deoxynivalenol by lactic acid bacteria and *bacillus* spp. Microorganisms 12 (9), 1892. 10.3390/microorganisms12091892 PMC11434589 39338565

[B55] Moyano-López C. Bridgeman L. Juan C. Juan-García A. (2025). Cytotoxic profiles of beauvericin, citrinin, moniliformin, and patulin and their binary combinations: a literature-based comparison and experimental validation in SH-SY5Y cells. Toxins 17 (3), 143. 10.3390/toxins17030143 PMC11945837 40137916

[B56] Niaz W. Iqbal S. Z. Ahmad K. Majid A. Haider W. Li X. (2025). Mycotoxins: a comprehensive review of its global trends in major cereals, advancements in chromatographic detections and future prospectives. Food. Chem. X 27, 102350. 10.1016/j.fochx.2025.102350 PMC11984607 40213337

[B57] Pestka J. J. (2010). Deoxynivalenol-induced proinflammatory gene expression: mechanisms and pathological sequelae. Toxins 2 (6), 1300–1317. 10.3390/toxins2061300 22069639 PMC3153246

[B58] Prosperini A. Juan-García A. Font G. Ruiz M. J. (2013). Reactive oxygen species involvement in apoptosis and mitochondrial damage in Caco-2 cells induced by enniatins A, A1, B and B1. Toxicol. Lett 222(1), 36–44. 10.1016/j.toxlet.2013.07.009 23867914

[B59] Qu Z. Ren X. Du Z. Hou J. Li Y. Yao Y. (2024). Fusarium mycotoxins: the major food contaminants. mLife 3 (2), 176–206. 10.1002/mlf2.12112 PMC11211685 38948146

[B60] Ribeiro E. Ladeira C. Viegas S. (2017). EDCs mixtures: a stealthy hazard for human health? Toxics 5 (1), 5. 10.3390/toxics5010005 29051438 PMC5606671

[B61] Sharma D. Asrani R. Ledoux D. Rottinghaus G. Gupta V. (2012). Toxic interaction between fumonisin B1 and moniliformin for cardiac lesions in Japanese quail. Avian Diseases 56, 545–554. 10.1637/10036-121111-Reg.1 23050472

[B62] Shen K. Tang Y. Shi J. Hu Z. He M. Li J. (2025). Relationship between aquatic fungal diversity in surface water and environmental factors in Yunnan dashanbao black-necked crane National nature reserve, China. J. Fungi 11 (7), 526. 10.3390/jof11070526 PMC12299766 40985445

[B63] Singh K. Kumari S. Singh B. Choubey R. B. Mitra D. K. Rai A. K. (2022). Jurkat T cells are immunophenotypically distinct from T-Cell acute lymphoblastic leukemia cells due to high-level surface expression of CD5. Cancer Investig. 40, 675–679. 10.1080/07357907.2022.2095397 35758332

[B64] Snezhkina A. V. Kudryavtseva A. V. Kardymon O. L. Savvateeva M. V. Melnikova N. V. Krasnov G. S. (2019). ROS generation and antioxidant defense systems in normal and malignant cells. Oxidative medicine Cellular Longevity 2019, 6175804. 10.1155/2019/6175804 31467634 PMC6701375

[B65] Søderstrøm S. Lie K. K. Lundebye A. K. Søfteland L. (2022). Beauvericin (BEA) and enniatin B (ENNB)-induced impairment of mitochondria and lysosomes - potential sources of intracellular reactive iron triggering ferroptosis in Atlantic salmon primary hepatocytes. Food Chemical toxicology an International Journal published Br. Industrial Biol. Res. Assoc. 161, 112819. 10.1016/j.fct.2022.112819 35038498

[B66] Sun X. Yang R. Tang H. Ma M. Chen H. Chang X. (2025). Diversity and pathogenicity of fusarium species associated with fusarium head blight in wheat and maize cropping systems in Sichuan Province. Sci. Rep. 15 (1), 5984. 10.1038/s41598-024-83402-7 PMC11836280 39966545

[B67] Szőke Z. Ruff E. Plank P. Molnár Z. Hruby L. Szentirmay A. (2025). Mycotoxin-induced oxidative stress and its impact on human folliculogenesis: examining the link to reproductive health. Toxins 17 (12), 574. 10.3390/toxins17120574 PMC12865479 41441610

[B68] Tonshin A. A. Teplova V. V. Andersson M. A. Salkinoja-Salonen M. S. (2010). The fusarium mycotoxins enniatins and beauvericin cause mitochondrial dysfunction by affecting the mitochondrial volume regulation, oxidative phosphorylation and ion homeostasis. Toxicology 276 (1), 49–57. 10.1016/j.tox.2010.07.001 20621153

[B69] Uhlig S. Torp M. Jarp J. Parich A. Gutleb § A. C. Krska R. (2004). Moniliformin in Norwegian grain. Food. Addit. and Contam. 21 (6), 598–606. 10.1080/02652030410001704258 15204539

[B80] Uhlig S. Eriksen G. S. Hofgaard I. S. Krska R. Beltran E. Sulyok M. (2013). Faces of a changing climate: semi-quantitative multi-mycotoxin analysis of grain grown in exceptional climatic conditions in Norway. Toxins (Basel) 5 (10), 1682–1697. 10.3390/toxins5101682 24084167 PMC3813906

[B70] Vilela R. S. Pina-Martins F. Ventura C. (2025). From food to humans: the toxicological effects of *Alternaria* mycotoxins in the liver and Colon. J. Xenobiotics 15 (6), 205. 10.3390/jox15060205 PMC12734068 41440752

[B71] Wadhwa K. Kapoor N. Kaur H. Abu-Seer E. A. Tariq M. Siddiqui S. (2024). A comprehensive review of the diversity of fungal secondary metabolites and their emerging applications in healthcare and environment. Mycobiology 52 (6), 335–387. 10.1080/12298093.2024.2416736 PMC11749308 39845176

[B72] Wattanasuntorn P. Poapolathep S. Phuektes P. Alassane-Kpembi I. Fink-Gremmels J. Oswald I. P. (2025). Apoptotic effect of combinations of T-2, HT-2, and diacetoxyscirpenol on human jurkat T cells. Toxins 17 (4), 203. 10.3390/toxins17040203 PMC12030997 40278701

[B73] Willoquet B. Mirey G. Labat O. Garofalo M. Puel S. Penary M. (2024). Roles of cytochromes P450 and ribosome inhibition in the interaction between two preoccupying mycotoxins, aflatoxin B1 and deoxynivalenol. Sci. Total Environ. 955, 176937. 10.1016/j.scitotenv.2024.176937 39437909

[B74] Wilson A. Dasyam N. O'Hagan F. Farrell D. Turner C. Jay A. (2025). Jurkat T-cell lines exhibit marked genomic instability affecting karyotype, mutational profile, gene expression, immunophenotype and function. Sci. Rep. 15 (1), 22426. 10.1038/s41598-025-95903-0 PMC12214745 40596466

[B75] Xia Y. Gao B. Zhang X. (2022). Targeting mitochondrial quality control of T cells: regulating the immune response in HCC. Front. Oncol. 12, 993437. 10.3389/fonc.2022.993437 PMC9539266 36212470

[B76] Yilmaz N. Verheecke-Vaessen C. Ezekiel C. N. (2025). Mycotoxins: an ongoing challenge to food safety and security. PLoS Pathog. 21 (11), e1013672. 10.1371/journal.ppat.1013672 PMC12599949 41212873

[B77] Yoshinari T. Takeuchi H. Taniguchi M. Fukumitsu T. Sato E. Zama S. (2025). Study about beauvericin and enniatins: method validation and survey for foods in Japan. Food Safety (Tokyo, Japan) 13 (4), 91–100. 10.14252/foodsafetyfscj.D-25-00018 PMC12718107 41426230

[B78] Zhang Y. Wang X. Liu X. Li Y. Feng D. Kang S. (2025). Optimization and metabolite profiling of mycotoxin enniatin B biodegradation by *Bacillus tequilensis* . Agronomy 15 (3), 522. 10.3390/agronomy15030522

[B79] Zhu Z. Cheng H. Wang J. Ma J. Wang J. Wang H. (2025). Occurrence of Co-Contamination and interaction of multi-mycotoxins in dairy cow feed in China. Toxins 17 (3), 137. 10.3390/toxins17030137 PMC11945585 40137910

