# Supplementary material for: Combined exposure to emerging Fusarium mycotoxins produces concentration-dependent synergistic toxicity in Jurkat T cells
Source: Front Toxicol. 2026 Sep 23;8:1924250. doi: 10.3389/ftox.2026.1924250 (PMC13641735; doi:10.3389/ftox.2026.1924250)
Supplement: Supplementary file 1 [file DataSheet1.pdf]

CompuSyn Report

**Experiment Name:**	MTT BEA ENN MON
**Date:**	1.12.25
**File Name:**	C:\Users\user\OneDrive - Universite de Montreal\Documents\Postdoc\Poapolathep\2025\BEA ENN MON_01.12.25.cse
**Description**	

Drug: BEA (BEA) [μM]
Drug: ENN B (ENNB) [μM]
Drug: MON (MON) [μM]
Drug Combo: BEA-ENNB (BEAENN) (BEA+ENNB [0.45:1.12])
Drug Combo: BEA-MON (BEAMON) (BEA+MON [0.45:10.11])
Drug Combo: ENNB-MON (ENNMON) (ENNB+MON [1.12:10.11])
Drug Combo: BEA-ENNB-MON (BEENMO) (BEA+ENNB+MON [0.45:1.12:10.11])

Data for Drug: BEA [μM]

Dose	Effect
0.45	0.25
0.91	0.42
1.81	0.5
3.62	0.59
7.24	0.79

5 data points entered.

X-int: 0.22856
Y-int: -0.1824 +/- 0.04549
m: 0.79796 +/- 0.09136
Dm: 1.69261
r: 0.98090

Data for Drug: ENNB [μM]

Dose	Effect
1.12	0.19
2.24	0.31
4.47	0.48
8.94	0.56
17.88	0.75

5 data points entered.

X-int: 0.74774
Y-int: -0.6628 +/- 0.04581
m: 0.88645 +/- 0.05893
Dm: 5.59422
r: 0.99344

Data for Drug: MON [μM]

Dose	Effect
10.11	0.08

Dose	Effect
20.23	0.29
40.45	0.48
80.9	0.53
161.8	0.67

5 data points entered.

**X-int:**	1.82000
**Y-int:**	-1.9211 +/- 0.33757
**m:**	1.05557 +/- 0.20307
**Dm:**	66.0686
**r:**	0.94872

Data for Drug Combo: BEAENN (BEA+ENNB [0.45:1.12])

Dose A	Effect
0.45+	0.48
0.91000+	0.51
1.81000+	0.62
3.62001+	0.74
7.24001+	0.8

5 data points entered.

**X-int:**	0.35925
**Y-int:**	-0.2039 +/- 0.05496
**m:**	0.56745 +/- 0.06062
**Dm:**	2.28692
**r:**	0.98331

Data for Drug Combo: BEAMON (BEA+MON [0.45:10.11])

Dose A	Effect
0.45000+	0.44
0.90085+	0.49
1.81000+	0.57
3.62000+	0.66
7.24001+	0.74

5 data points entered.

**X-int:**	1.31242
**Y-int:**	-0.6191 +/- 0.05292
**m:**	0.47175 +/- 0.03146
**Dm:**	20.5313
**r:**	0.99340

Data for Drug Combo: ENNMON (ENNB+MON [1.12:10.11])

Dose A	Effect
1.12+	0.42
2.24+	0.48
4.47000+	0.52
8.94000+	0.63
17.8800+	0.7

5 data points entered.

X-int: 1.43659
Y-int: -0.6125 +/- 0.06827
m: 0.42638 +/- 0.04002
Dm: 27.3267
r: 0.98704

Data for Drug Combo: BEENMO (BEA+ENNB+MON [0.45:1.12:10.11])

Dose A	Effect
0.45+	0.61
0.91000+	0.69
1.81000+	0.77
3.62000+	0.82
7.23999+	0.92

5 data points entered.

X-int: 0.85050
Y-int: -0.5769 +/- 0.15838
m: 0.67829 +/- 0.09180
Dm: 7.08757
r: 0.97361

Dose-Effect Curve for Drugs

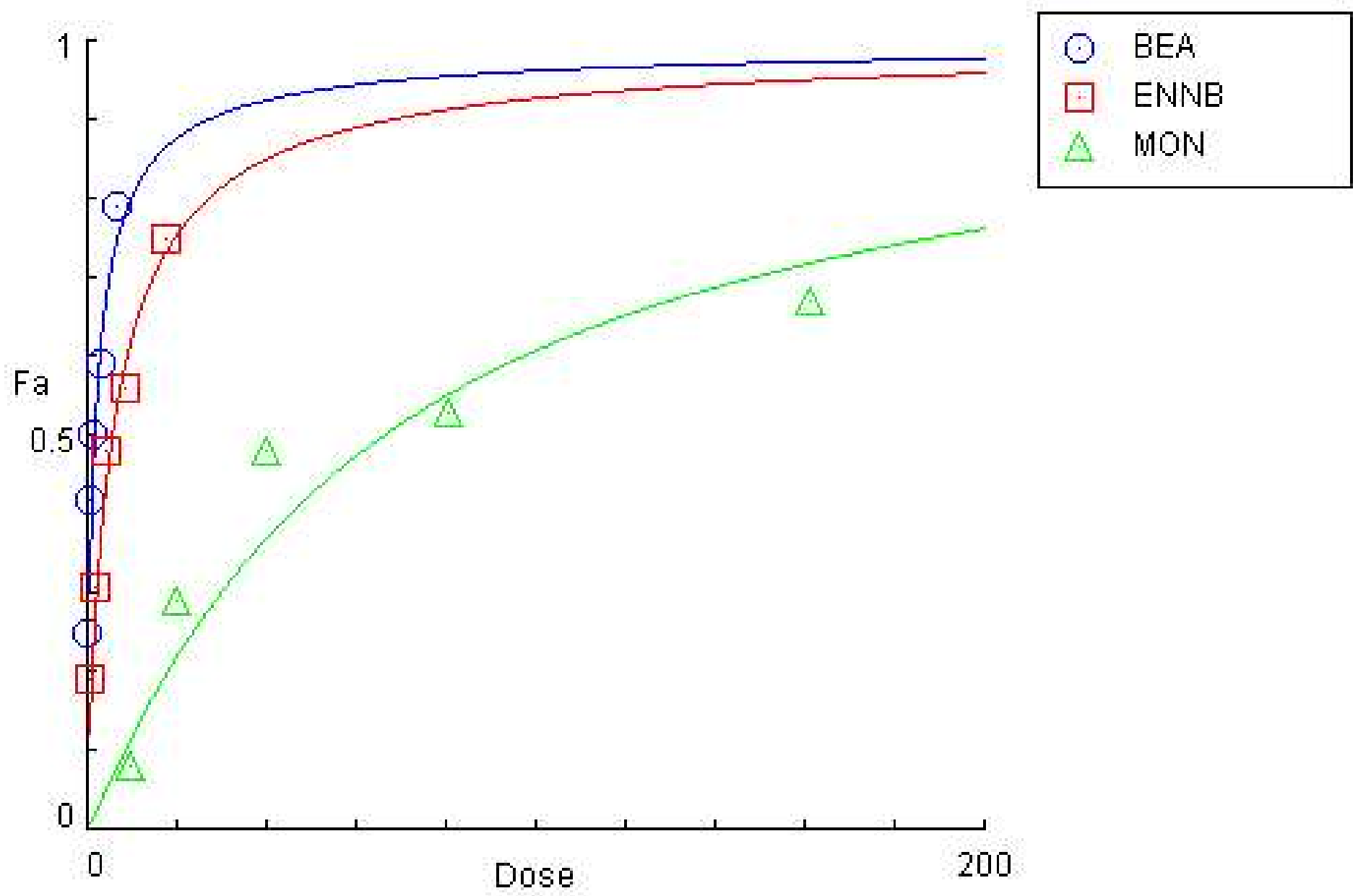

Dose-Effect Curve for Drug Combos

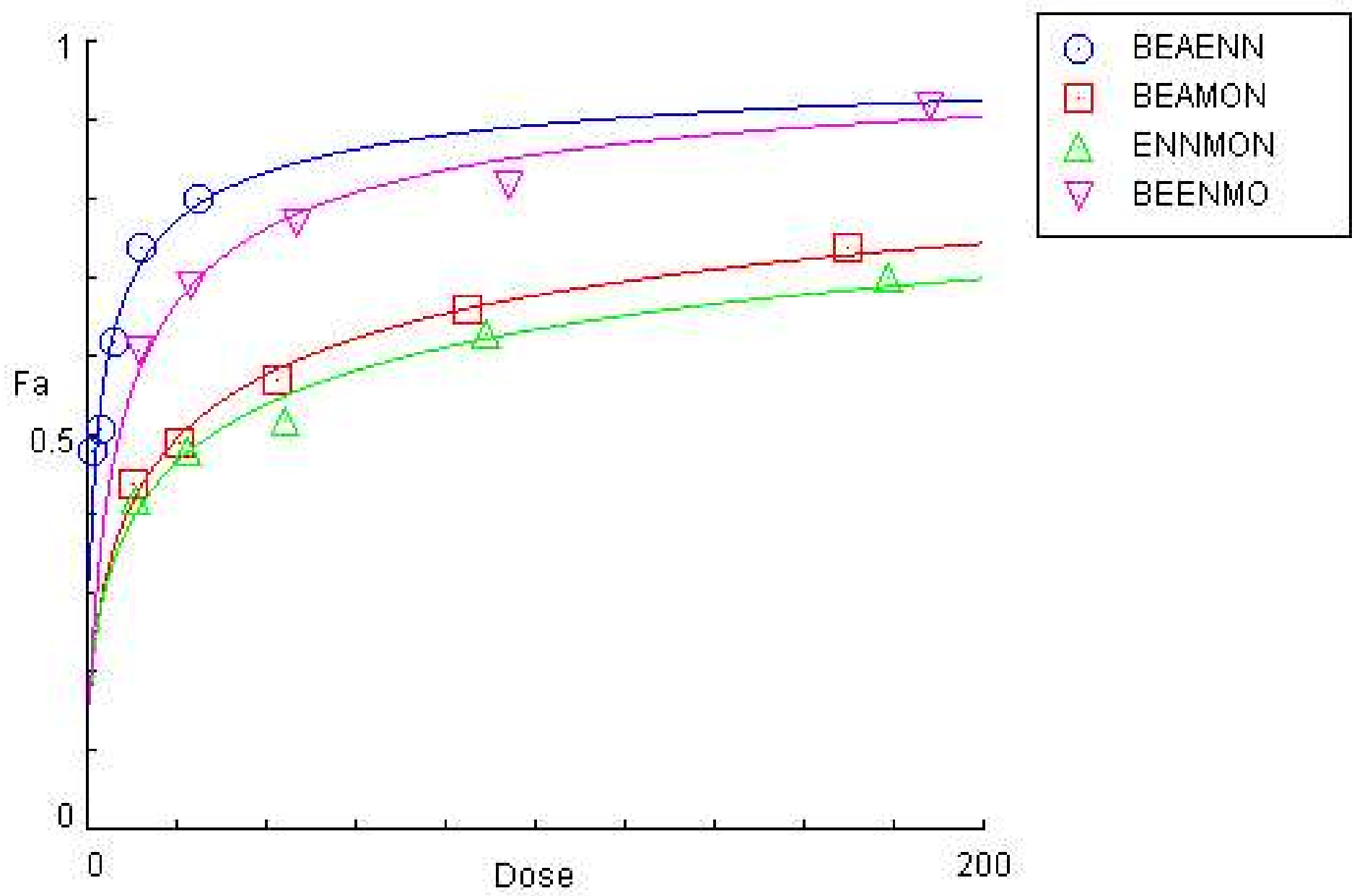

Median-Effect Plot for Drugs

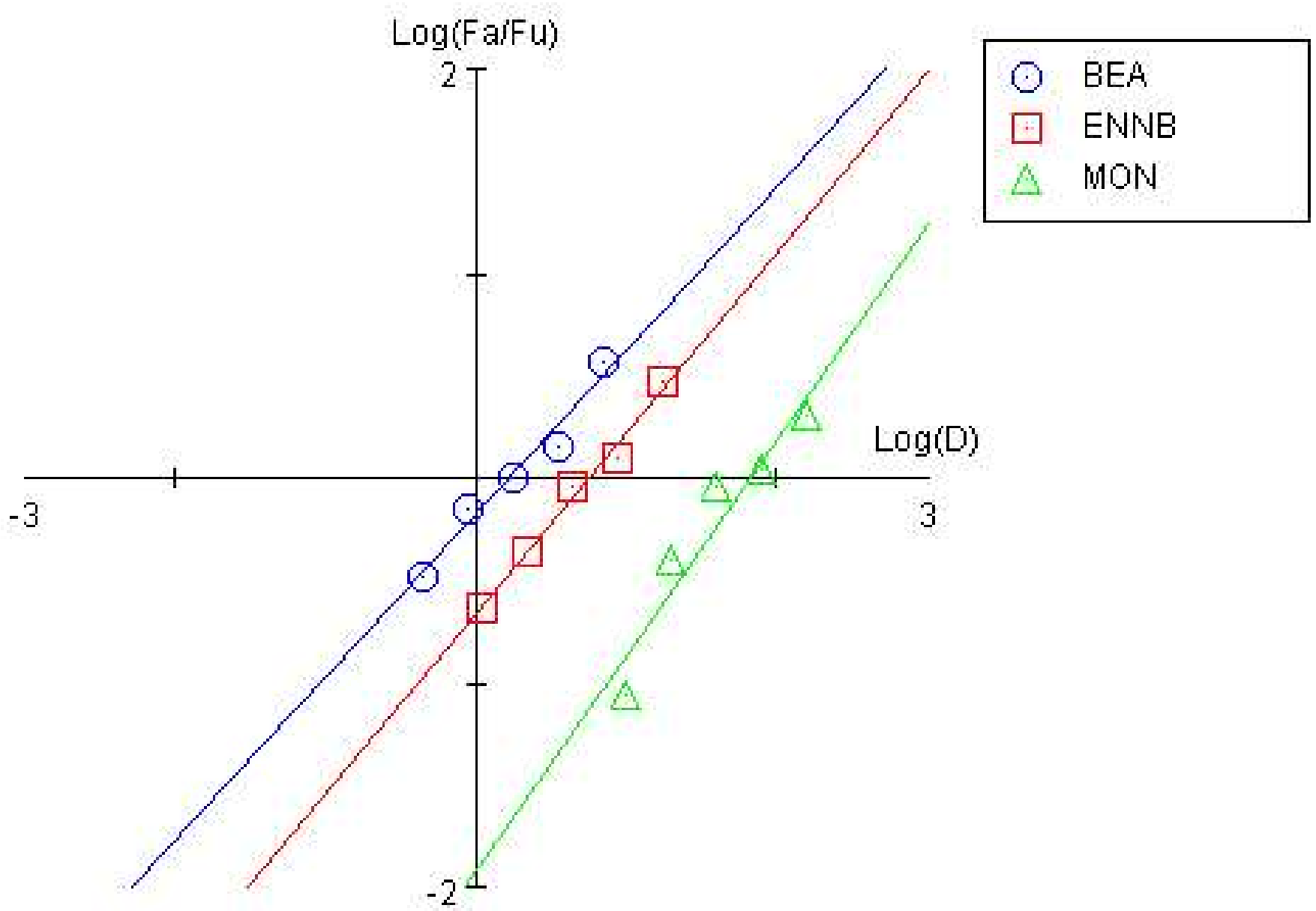

Median-Effect Plot for Drug Combos

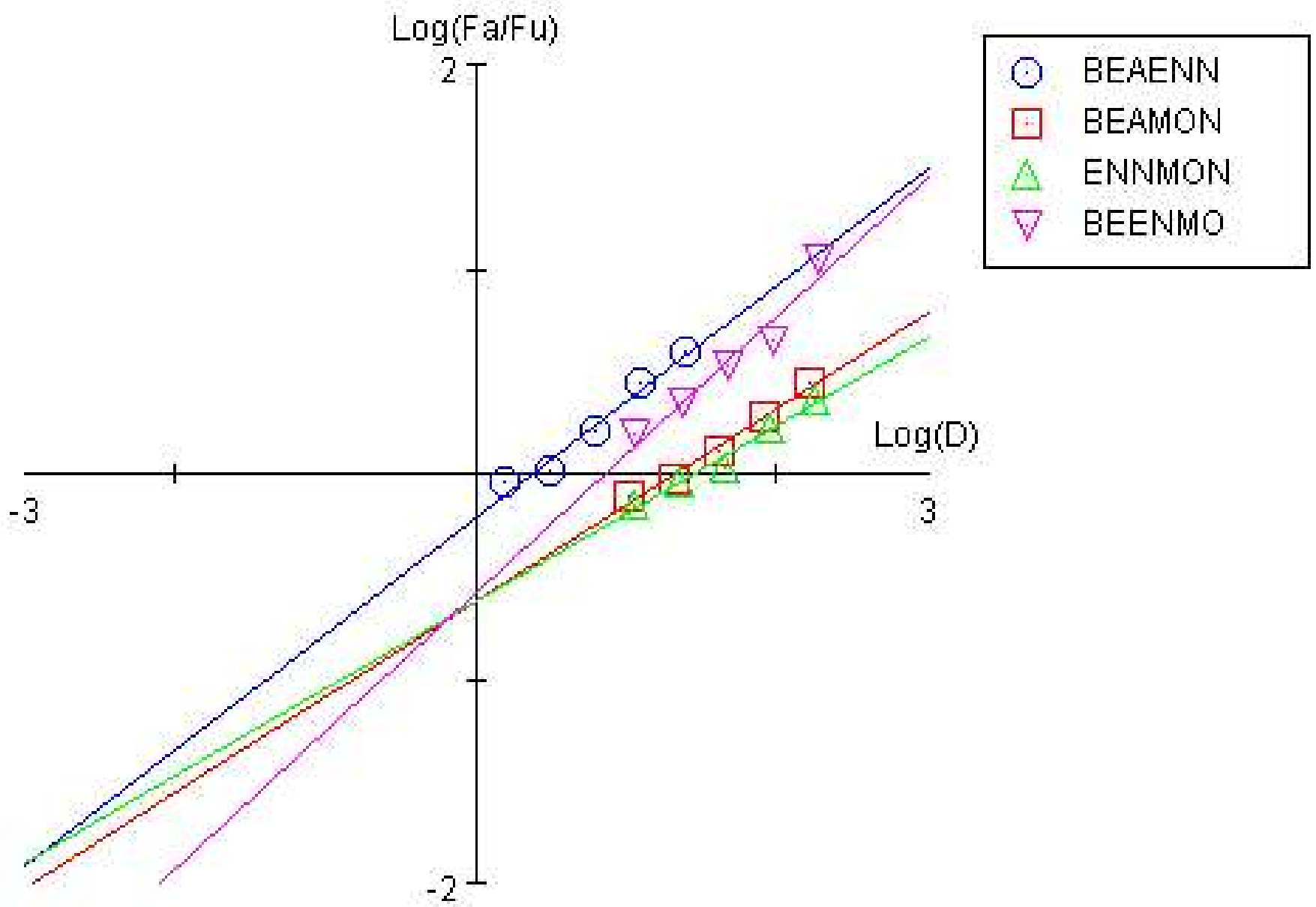

CI Data for Drug Combo: BEAENN (BEA+ENNB [0.45:1.12])

Fa	CI Value	S.D.A. Analysis
0.05	0.13157	0.14257 +/- 0.11389
0.1	0.19892	0.20975 +/- 0.12509
0.15	0.25721	0.26748 +/- 0.12941
0.2	0.31227	0.32184 +/- 0.13050
0.25	0.36666	0.37543 +/- 0.12951
0.3	0.42198	0.42987 +/- 0.12694
0.35	0.47952	0.48647 +/- 0.12302
0.4	0.54054	0.54646 +/- 0.11792
0.45	0.60643	0.61124 +/- 0.11178
0.5	0.67889	0.68247 +/- 0.10492
0.55	0.76013	0.76235 +/- 0.09807
0.6	0.85319	0.85391 +/- 0.09290
0.65	0.96258	0.96160 +/- 0.09298
0.7	1.09524	1.09232 +/- 0.10438
0.75	1.26284	1.25766 +/- 0.13462
0.8	1.48685	1.47900 +/- 0.19272
0.85	1.81268	1.80165 +/- 0.29668
0.9	2.36012	2.34559 +/- 0.49936
0.95	3.62095	3.60600 +/- 1.04066
0.97	4.91658	4.91049 +/- 1.66526

CI values for actual experimental points:

Total Dose	Fa	CI Value
1.57	0.48	0.51304
3.17489	0.51	0.89834
6.31489	0.62	1.04256
12.6298	0.74	1.07152
25.2596	0.8	1.42707

CI Data for Drug Combo: BEAMON (BEA+MON [0.45:10.11])

Fa	CI Value	S.D.A. Analysis
0.05	0.04972	0.05384 +/- 0.03417
0.1	0.09963	0.10499 +/- 0.04966
0.15	0.15391	0.15993 +/- 0.06064
0.2	0.21405	0.22042 +/- 0.06873
0.25	0.28156	0.28805 +/- 0.07449
0.3	0.35822	0.36464 +/- 0.07812
0.35	0.44628	0.45246 +/- 0.07975
0.4	0.54871	0.55450 +/- 0.07966
0.45	0.66954	0.67479 +/- 0.07876
0.5	0.81441	0.81898 +/- 0.07969
0.55	0.99150	0.99525 +/- 0.08847
0.6	1.21310	1.21594 +/- 0.11460
0.65	1.49860	1.50055 +/- 0.16842
0.7	1.88055	1.88184 +/- 0.26294
0.75	2.41791	2.41940 +/- 0.42319
0.8	3.22976	3.23396 +/- 0.70574
0.85	4.59743	4.61224 +/- 1.25798
0.9	7.38064	7.43670 +/- 2.58008
0.95	16.0212	16.3261 +/- 7.66502
0.97	28.0255	28.8780 +/- 16.1341

CI values for actual experimental points:

Total Dose	Fa	CI Value
10.56	0.44	0.55197
21.14	0.49	0.87776
42.4747	0.57	1.22240
84.9493	0.66	1.58811
169.899	0.74	2.06720

CI Data for Drug Combo: ENNMON (ENNB+MON [1.12:10.11])

Fa	CI Value	S.D.A. Analysis
0.05	0.01960	0.02102 +/- 0.01323
0.1	0.05084	0.05321 +/- 0.02464
0.15	0.09193	0.09505 +/- 0.03490
0.2	0.14374	0.14748 +/- 0.04401
0.25	0.20808	0.21233 +/- 0.05184
0.3	0.28760	0.29228 +/- 0.05811
0.35	0.38604	0.39109 +/- 0.06256
0.4	0.50865	0.51402 +/- 0.06511
0.45	0.66287	0.66854 +/- 0.06660
0.5	0.85954	0.86554 +/- 0.07077
0.55	1.11491	1.12139 +/- 0.08750
0.6	1.45439	1.46166 +/- 0.13173
0.65	1.91968	1.92846 +/- 0.22004
0.7	2.58371	2.59554 +/- 0.37907
0.75	3.58510	3.60355 +/- 0.66560
0.8	5.21940	5.25332 +/- 1.21419

Fa	CI Value	S.D.A. Analysis
0.85	8.23204	8.30730 +/- 2.40085
0.9	15.1010	15.3179 +/- 5.63342
0.95	40.3828	41.4695 +/- 20.7012
0.97	81.5337	84.7137 +/- 50.5204

CI values for actual experimental points:

Total Dose	Fa	CI Value
11.23	0.42	0.49590
22.46	0.48	0.76841
44.8197	0.52	1.29618
89.6395	0.63	1.61444
179.279	0.7	2.32361

CI Data for Drug Combo: BEENMO (BEA+ENNB+MON [0.45:1.12:10.11])

Fa	CI Value	S.D.A. Analysis
0.05	0.14764	0.15541 +/- 0.08983
0.1	0.18519	0.19119 +/- 0.09156
0.15	0.21380	0.21854 +/- 0.09150
0.2	0.23864	0.24239 +/- 0.09046
0.25	0.26163	0.26454 +/- 0.08864
0.3	0.28378	0.28597 +/- 0.08613
0.35	0.30575	0.30732 +/- 0.08290
0.4	0.32806	0.32909 +/- 0.07892
0.45	0.35120	0.35178 +/- 0.07410
0.5	0.37567	0.37590 +/- 0.06838
0.55	0.40209	0.40209 +/- 0.06178
0.6	0.43123	0.43115 +/- 0.05470
0.65	0.46419	0.46425 +/- 0.04871
0.7	0.50259	0.50314 +/- 0.04853
0.75	0.54906	0.55068 +/- 0.06256
0.8	0.60830	0.61202 +/- 0.09855
0.85	0.68985	0.69787 +/- 0.16854
0.9	0.81776	0.83587 +/- 0.31325
0.95	1.08423	1.13659 +/- 0.73724
0.97	1.33255	1.43303 +/- 1.27803

CI values for actual experimental points:

Total Dose	Fa	CI Value
11.68	0.61	0.37281
23.6196	0.69	0.50643
46.9796	0.77	0.63720
93.9591	0.82	0.90359
187.918	0.92	0.64872

Combination Index Plot

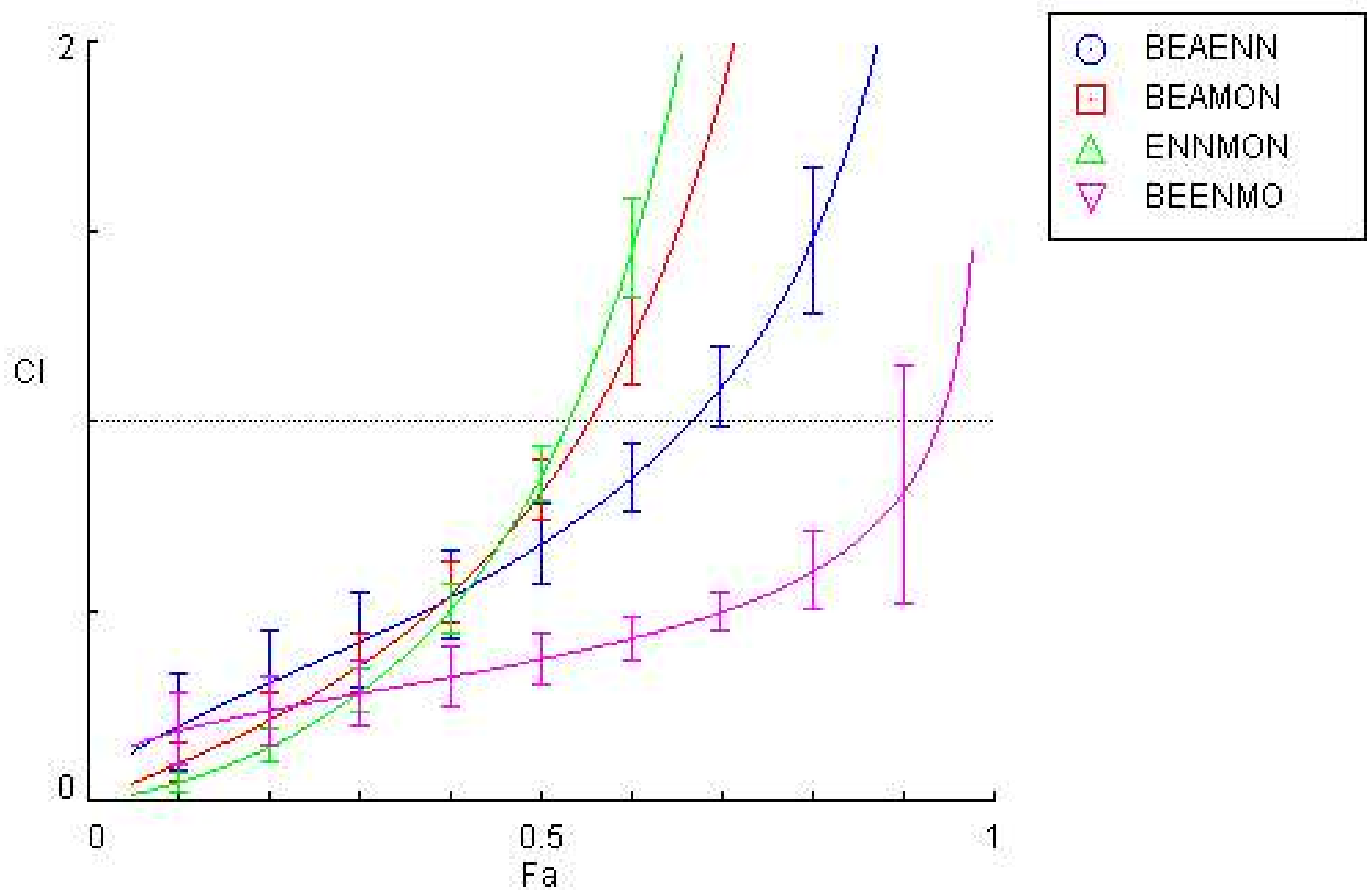

Logarithmic Combination Index Plot

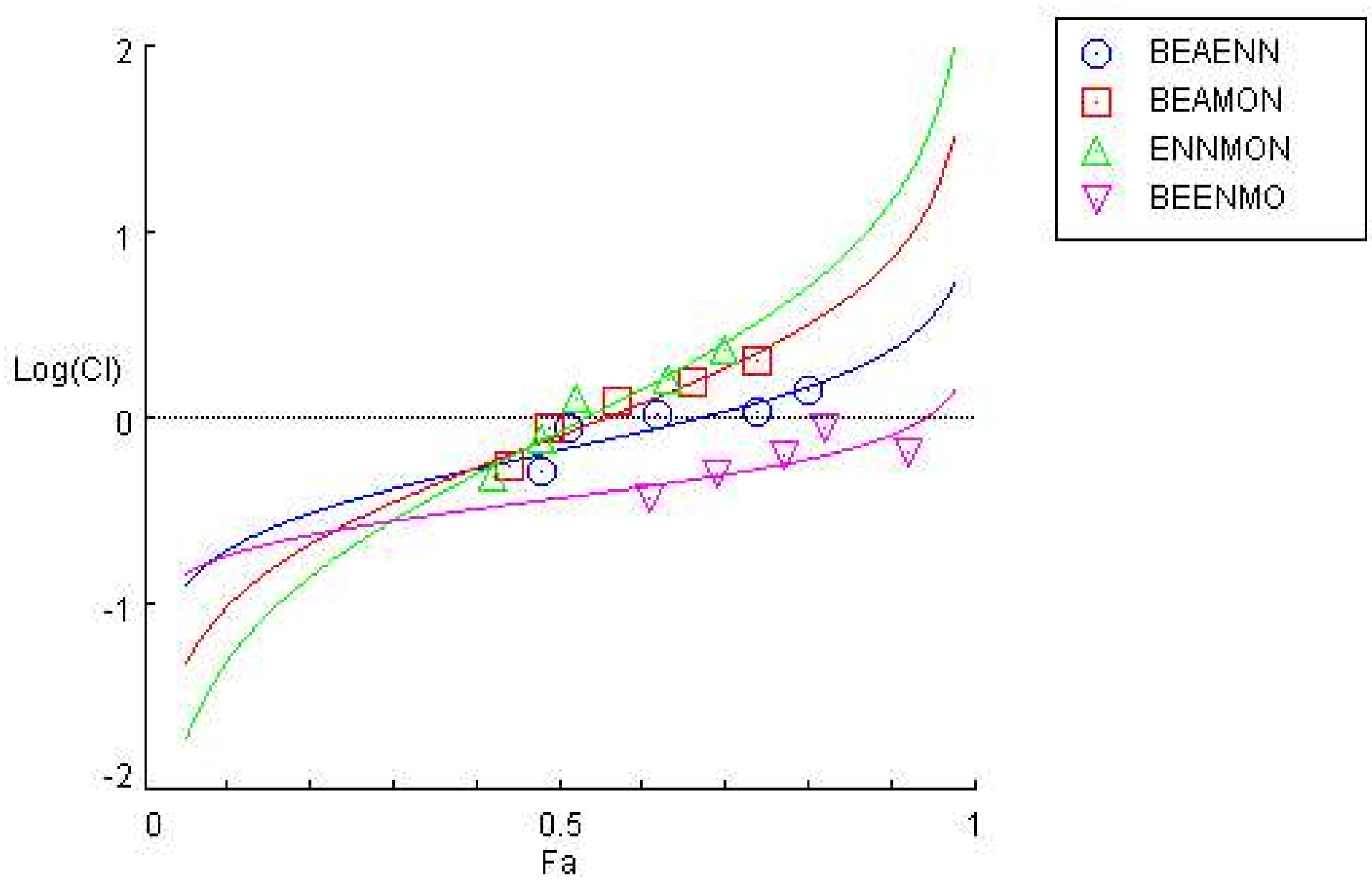

DRI Data for Drug Combo: BEAENN (BEA+ENNB [0.45:1.12])

Fa	Dose BEA	Dose ENNB	DRI BEA	DRI ENNB
0.05	0.04227	0.20192	11.5610	22.1890
0.1	0.10782	0.46910	7.90294	13.8146
0.15	0.19253	0.79053	6.24460	10.3020
0.2	0.29790	1.17101	5.22993	8.26015
0.25	0.42720	1.61996	4.51742	6.88262
0.3	0.58535	2.15094	3.97490	5.86861
0.35	0.77919	2.78264	3.53883	5.07771
0.4	1.01832	3.54073	3.17424	4.43450
0.45	1.31626	4.46094	2.85996	3.89439
0.5	1.69261	5.59422	2.58222	3.42902

Fa	Dose BEA	Dose ENNB	DRI BEA	DRI ENNB
0.55	2.17657	7.01541	2.33145	3.01926
0.6	2.81341	8.83866	2.10062	2.65152
0.65	3.67681	11.2466	1.88420	2.31564
0.7	4.89443	14.5496	1.67749	2.00356
0.75	6.70626	19.3186	1.47603	1.70838
0.8	9.61728	26.7250	1.27494	1.42348
0.85	14.8806	39.5878	1.06778	1.14135
0.9	26.5707	66.7132	0.84372	0.85114
0.95	67.7761	154.985	0.57676	0.52991
0.97	131.957	282.334	0.43999	0.37824

DRI values calculated at experimental points

Fa	Dose BEA	Dose ENNB	DRI BEA	DRI ENNB
0.48	1.53107	5.11122	3.40237	4.56359
0.51	1.77963	5.85247	1.95564	2.58400
0.62	3.12605	9.71809	1.72710	2.15723
0.74	6.27811	18.2047	1.73428	2.02055
0.8	9.61728	26.7250	1.32835	1.48311

DRI Data for Drug Combo: BEAMON (BEA+MON [0.45:10.11])

Fa	Dose BEA	Dose MON	DRI BEA	DRI MON
0.05	0.04227	4.06028	24.8152	106.095
0.1	0.10782	8.24108	12.9870	44.1815
0.15	0.19253	12.7739	8.69765	25.6856
0.2	0.29790	17.7676	6.43159	17.0743
0.25	0.42720	23.3340	5.01246	12.1862
0.3	0.58535	29.6066	4.03153	9.07625
0.35	0.77919	36.7538	3.30800	6.94520
0.4	1.01832	44.9959	2.74909	5.40681
0.45	1.31626	54.6302	2.30205	4.25272
0.5	1.69261	66.0686	1.93461	3.36118
0.55	2.17657	79.9020	1.62582	2.65655
0.6	2.81341	97.0101	1.36144	2.08951
0.65	3.67681	118.765	1.13141	1.62667
0.7	4.89443	147.435	0.92836	1.24474
0.75	6.70626	187.068	0.74668	0.92708
0.8	9.61728	245.676	0.58193	0.66167
0.85	14.8806	341.718	0.43031	0.43984
0.9	26.5707	529.671	0.28819	0.25571
0.95	67.7761	1075.06	0.15082	0.10649
0.97	131.957	1779.00	0.09514	0.05709

DRI values calculated at experimental points

Fa	Dose BEA	Dose MON	DRI BEA	DRI MON
0.44	1.25114	52.5742	2.78030	5.20022
0.49	1.60985	63.6115	1.78703	3.14299
0.57	2.40966	86.2895	1.33130	2.12198
0.66	3.88649	123.850	1.07362	1.52282
0.74	6.27811	177.968	0.86714	1.09412

DRI Data for Drug Combo: ENNMON (ENNB+MON [1.12:10.11])

Fa	Dose ENNB	Dose MON	DRI ENNB	DRI MON
0.05	0.20192	4.06028	73.9341	164.694
0.1	0.46910	8.24108	29.7743	57.9463
0.15	0.79053	12.7739	16.9544	30.3498
0.2	1.17101	17.7676	11.0958	18.6505
0.25	1.61996	23.3340	7.81768	12.4747
0.3	2.15094	29.6066	5.75738	8.77914
0.35	2.78264	36.7538	4.36073	6.38073
0.4	3.54073	44.9959	3.36246	4.73374
0.45	4.46094	54.6302	2.62057	3.55524
0.5	5.59422	66.0686	2.05265	2.68557
0.55	7.01541	79.9020	1.60780	2.02864
0.6	8.83866	97.0101	1.25306	1.52359
0.65	11.2466	118.765	0.96620	1.13032
0.7	14.5496	147.435	0.73182	0.82153
0.75	19.3186	187.068	0.53895	0.57815
0.8	26.7250	245.676	0.37973	0.38671
0.85	39.5878	341.718	0.24851	0.23764
0.9	66.7132	529.671	0.14151	0.12447
0.95	154.985	1075.06	0.05699	0.04379
0.97	282.334	1779.00	0.02984	0.02083

DRI values calculated at experimental points

Fa	Dose ENNB	Dose MON	DRI ENNB	DRI MON
0.42	3.88692	48.6626	3.47046	4.81332
0.48	5.11122	61.2439	2.28180	3.02888
0.52	6.12286	71.2734	1.36977	1.76639
0.63	10.1973	109.387	1.14064	1.35549
0.7	14.5496	147.435	0.81374	0.91348

DRI Data for Drug Combo: BEENMO (BEA+ENNB+MON [0.45:1.12:10.11])

Fa	Dose BEA	Dose ENNB	Dose MON	DRI BEA	DRI ENNB	DRI MON
0.05	0.04227	0.20192	4.06028	11.8862	22.8133	50.8183
0.1	0.10782	0.46910	8.24108	10.0760	17.6132	34.2785
0.15	0.19253	0.79053	12.7739	9.09627	15.0065	26.8628
0.2	0.29790	1.17101	17.7676	8.42201	13.3017	22.3584
0.25	0.42720	1.61996	23.3340	7.90296	12.0407	19.2135
0.3	0.58535	2.15094	29.6066	7.47578	11.0374	16.8303
0.35	0.77919	2.78264	36.7538	7.10783	10.1987	14.9230
0.4	1.01832	3.54073	44.9959	6.77997	9.47179	13.3346
0.45	1.31626	4.46094	54.6302	6.47980	8.82348	11.9705
0.5	1.69261	5.59422	66.0686	6.19856	8.23128	10.7694
0.55	2.17657	7.01541	79.9020	5.92954	7.67883	9.68872
0.6	2.81341	8.83866	97.0101	5.66702	7.15323	8.69760
0.65	3.67681	11.2466	118.765	5.40562	6.64339	7.77183
0.7	4.89443	14.5496	147.435	5.13956	6.13860	6.89107
0.75	6.70626	19.3186	187.068	4.86175	5.62707	6.03634

Fa	Dose BEA	Dose ENNB	Dose MON	DRI BEA	DRI ENNB	DRI MON
0.8	9.61728	26.7250	245.676	4.56212	5.09362	5.18726
0.85	14.8806	39.5878	341.718	4.22395	4.51498	4.31746
0.9	26.5707	66.7132	529.671	3.81324	3.84678	3.38344
0.95	67.7761	154.985	1075.06	3.23251	2.96994	2.28223
0.97	131.957	282.334	1779.00	2.87399	2.47063	1.72460

DRI values calculated at experimental points

Fa	Dose BEA	Dose ENNB	Dose MON	DRI BEA	DRI ENNB	DRI MON
0.61	2.96489	9.26592	100.933	6.58865	8.27314	9.98350
0.69	4.61344	13.7955	140.991	5.06970	6.09101	6.89620
0.77	7.69457	21.8635	207.555	4.25114	4.85328	5.10407
0.82	11.3197	30.9481	277.888	3.12698	3.43495	3.41683
0.92	36.1253	87.9636	668.124	4.98969	4.88157	4.10753

DRI Plot for Combo: BEAENN (BEA+ENNB [0.45:1.12])

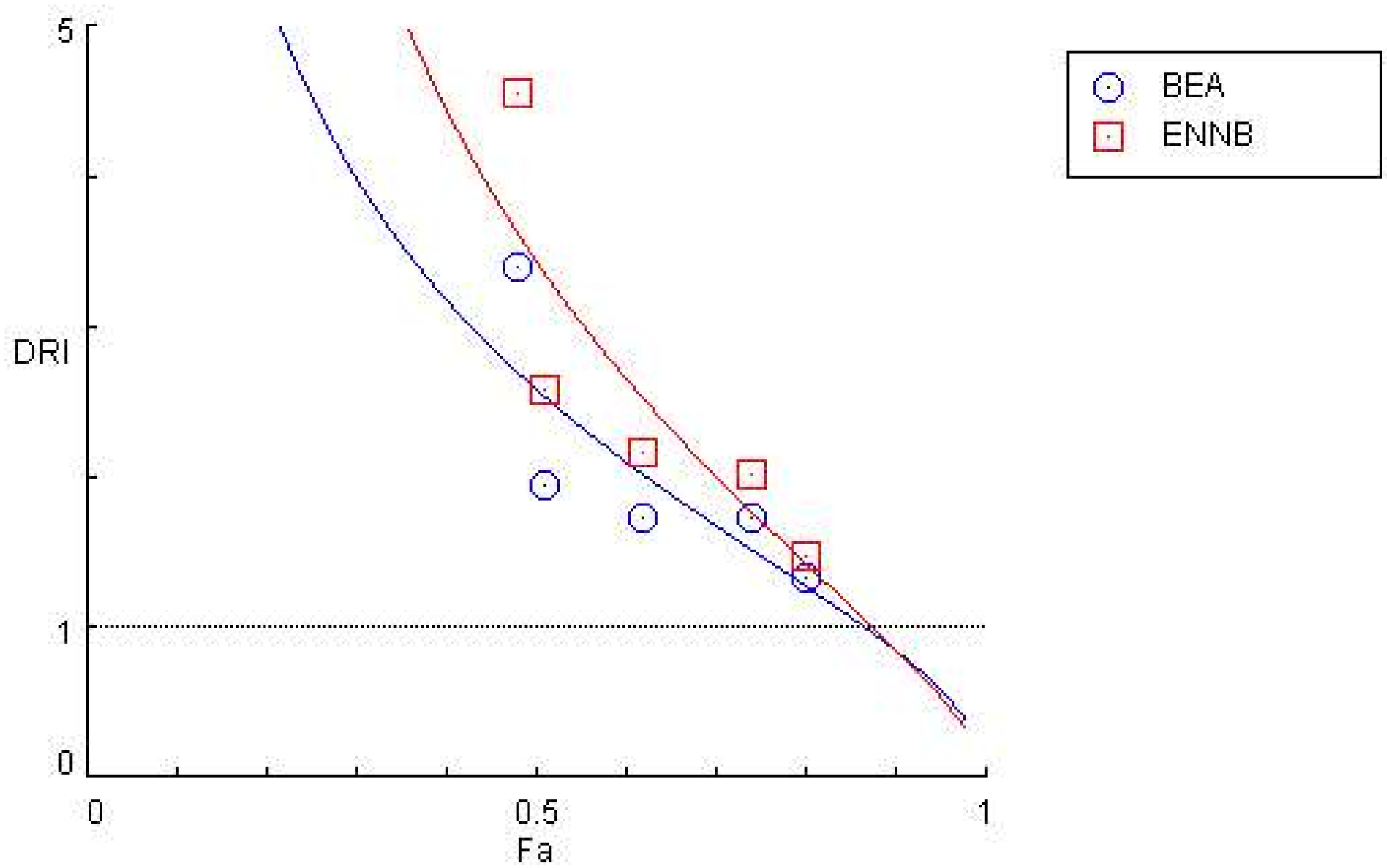

DRI Plot for Combo: BEAMON (BEA+MON [0.45:10.11])

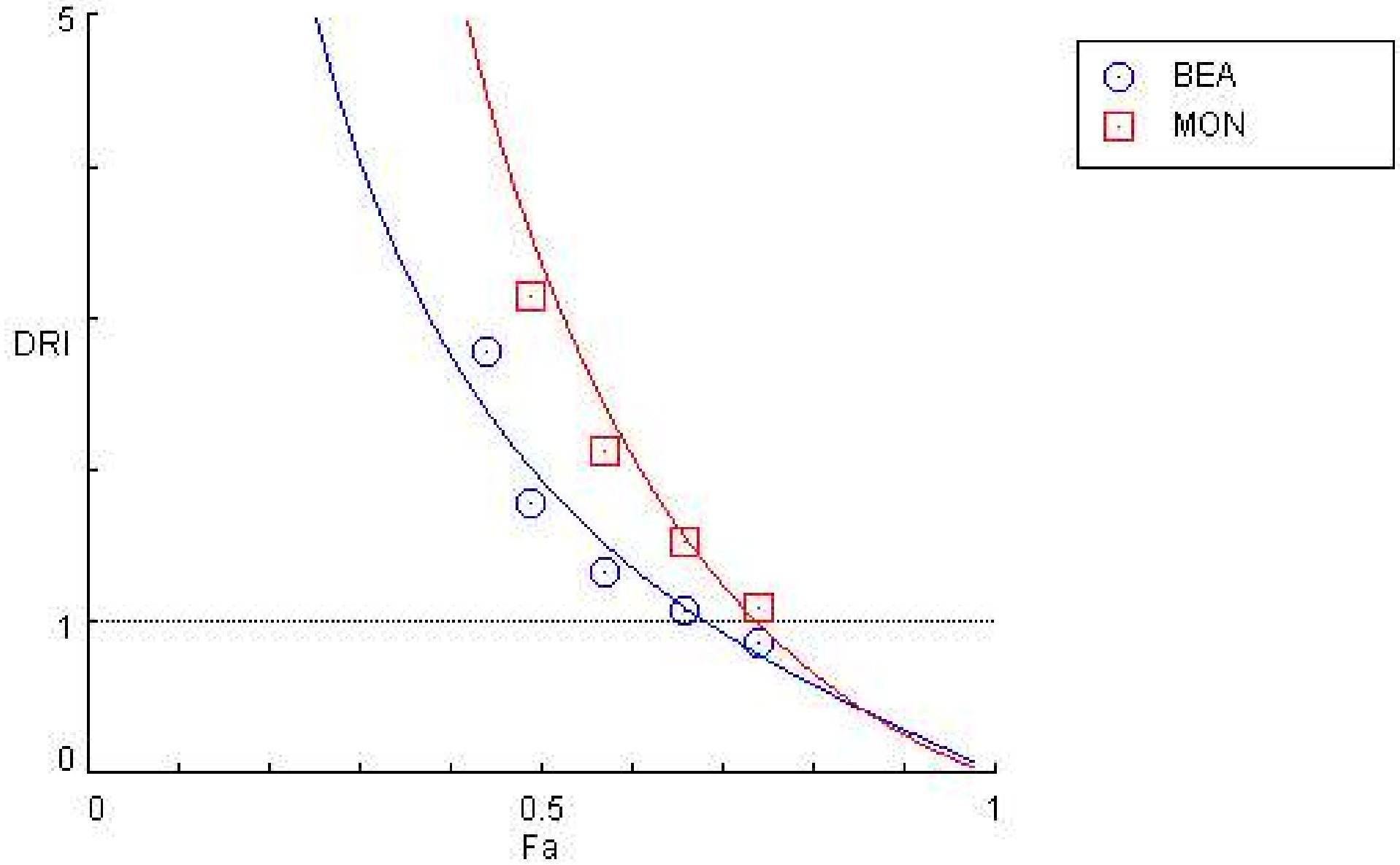

DRI Plot for Combo: ENNMON (ENNB+MON [1.12:10.11])

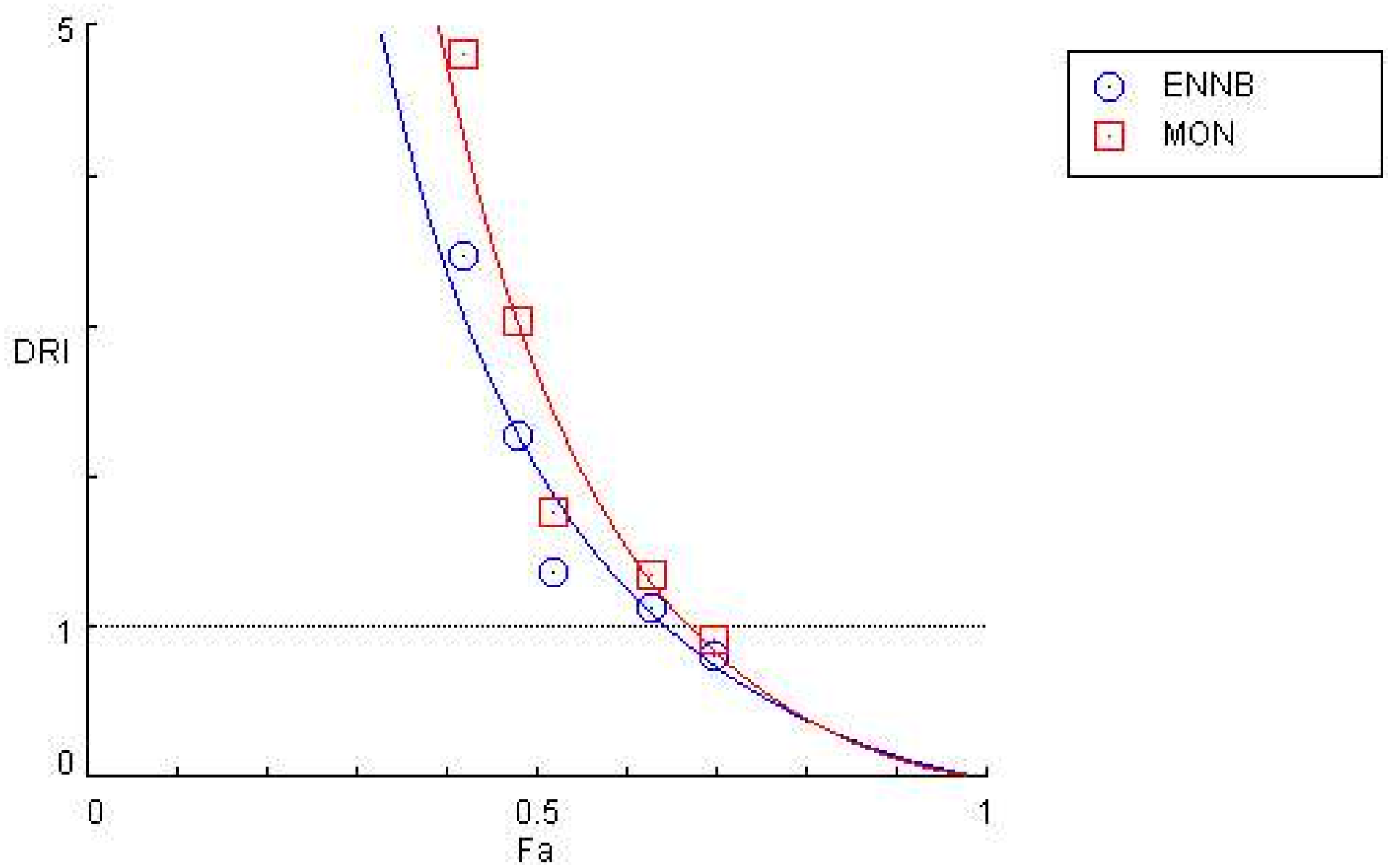

DRI Plot for Combo: BEENMO (BEA+ENNB+MON [0.45:1.12:10.11])

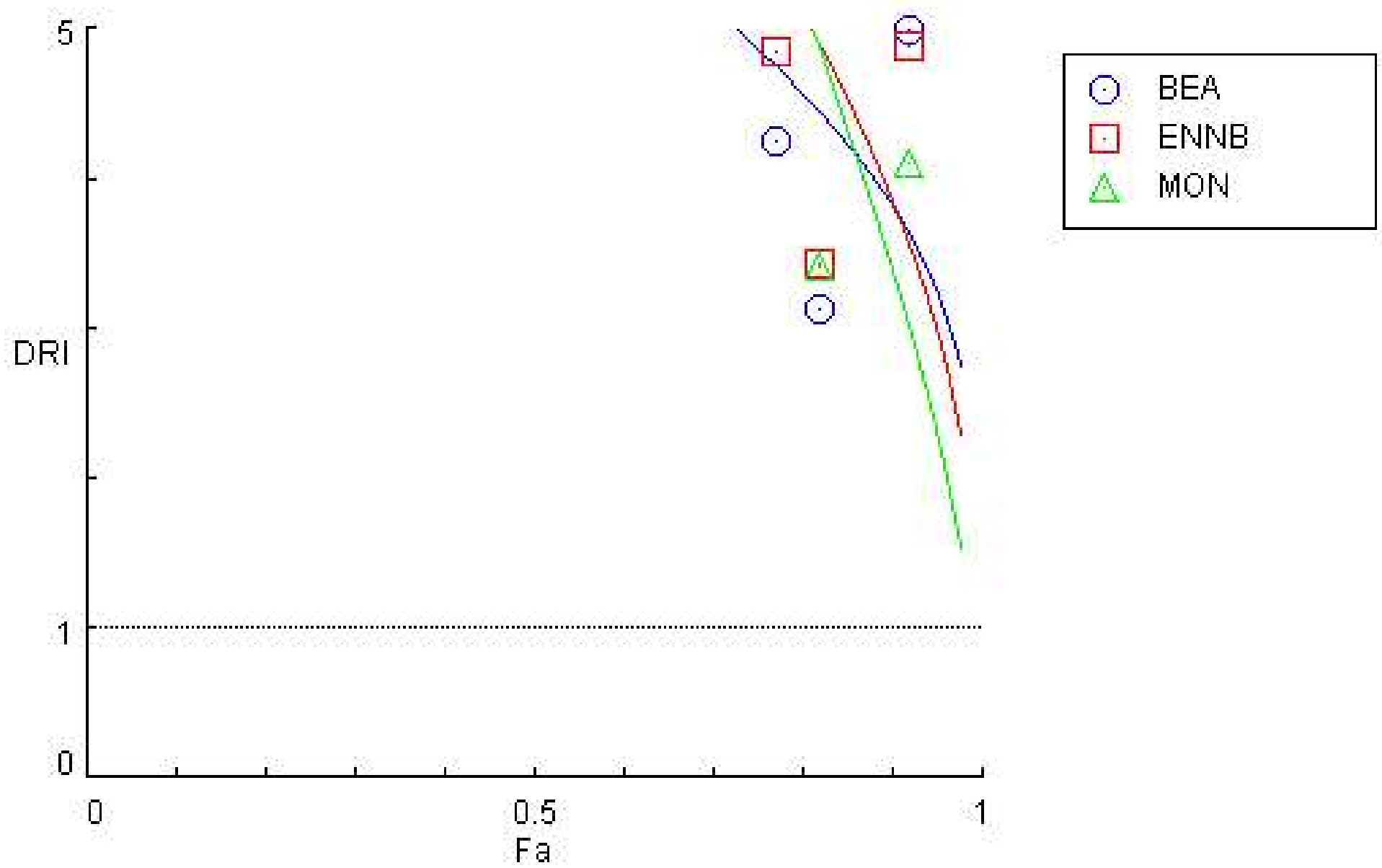

Log(DRI) Plot for Combo: BEAENN (BEA+ENNB [0.45:1.12])

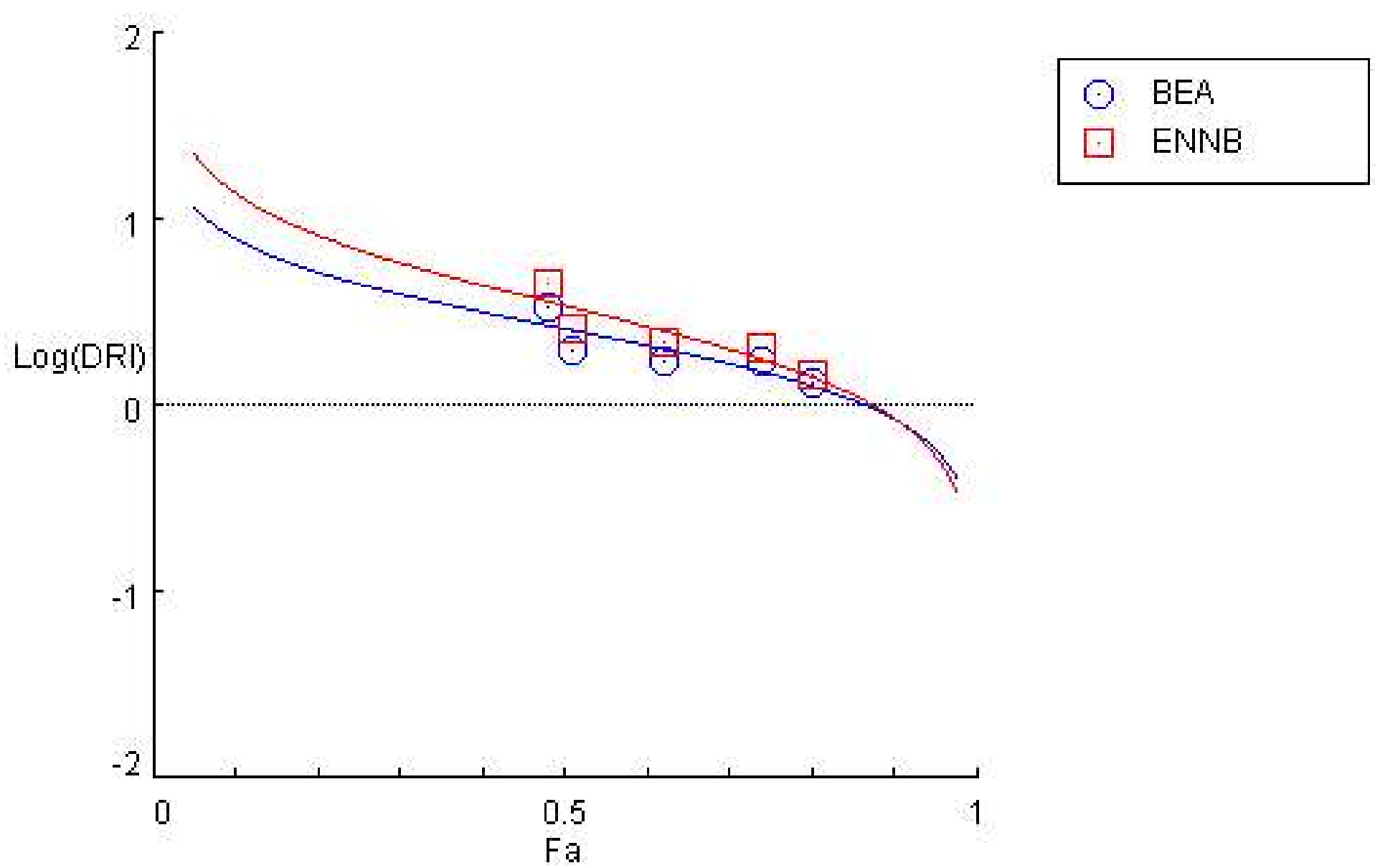

Log(DRI) Plot for Combo: BEAMON (BEA+MON [0.45:10.11])

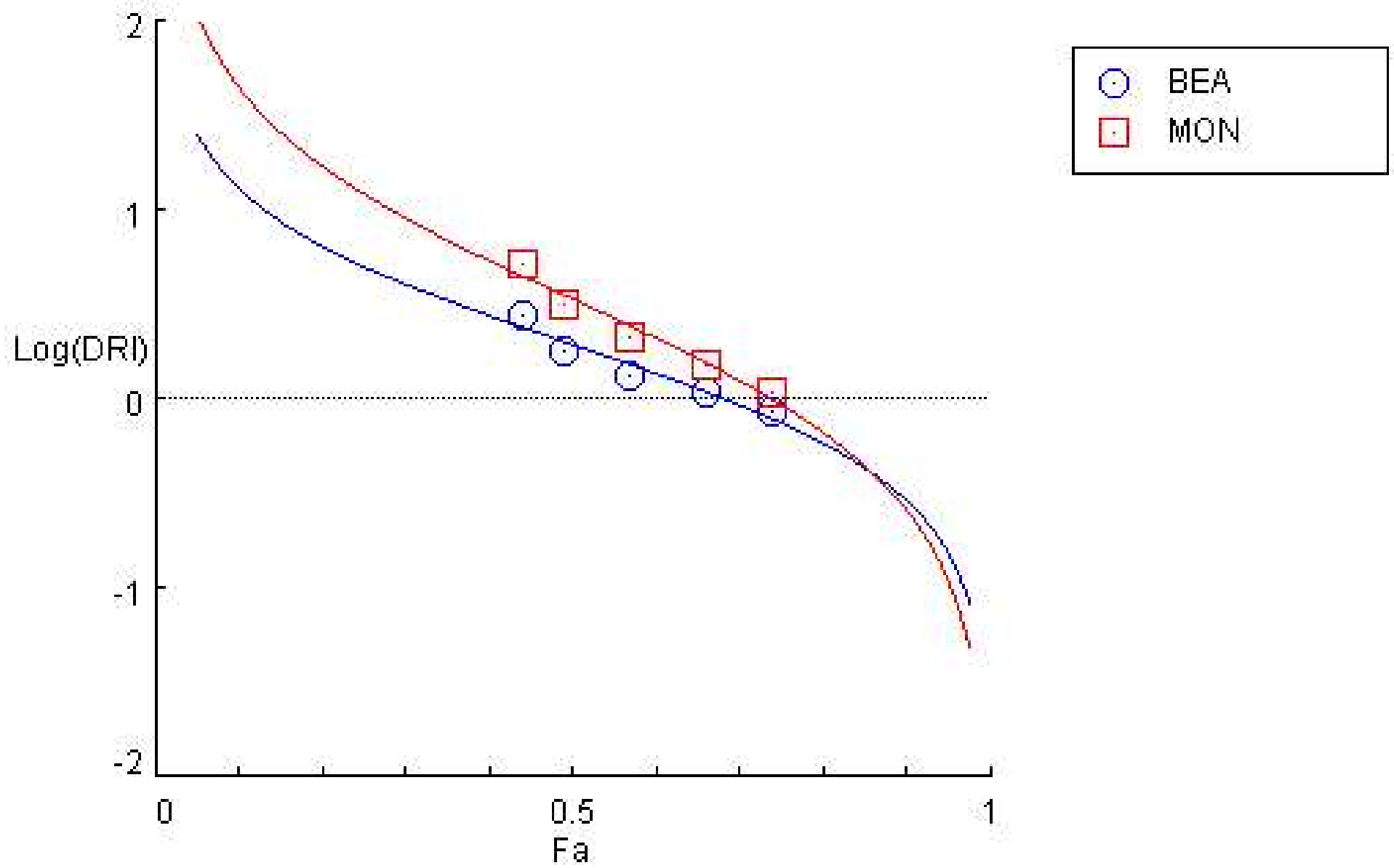

Log(DRI) Plot for Combo: ENNMON (ENNB+MON [1.12:10.11])

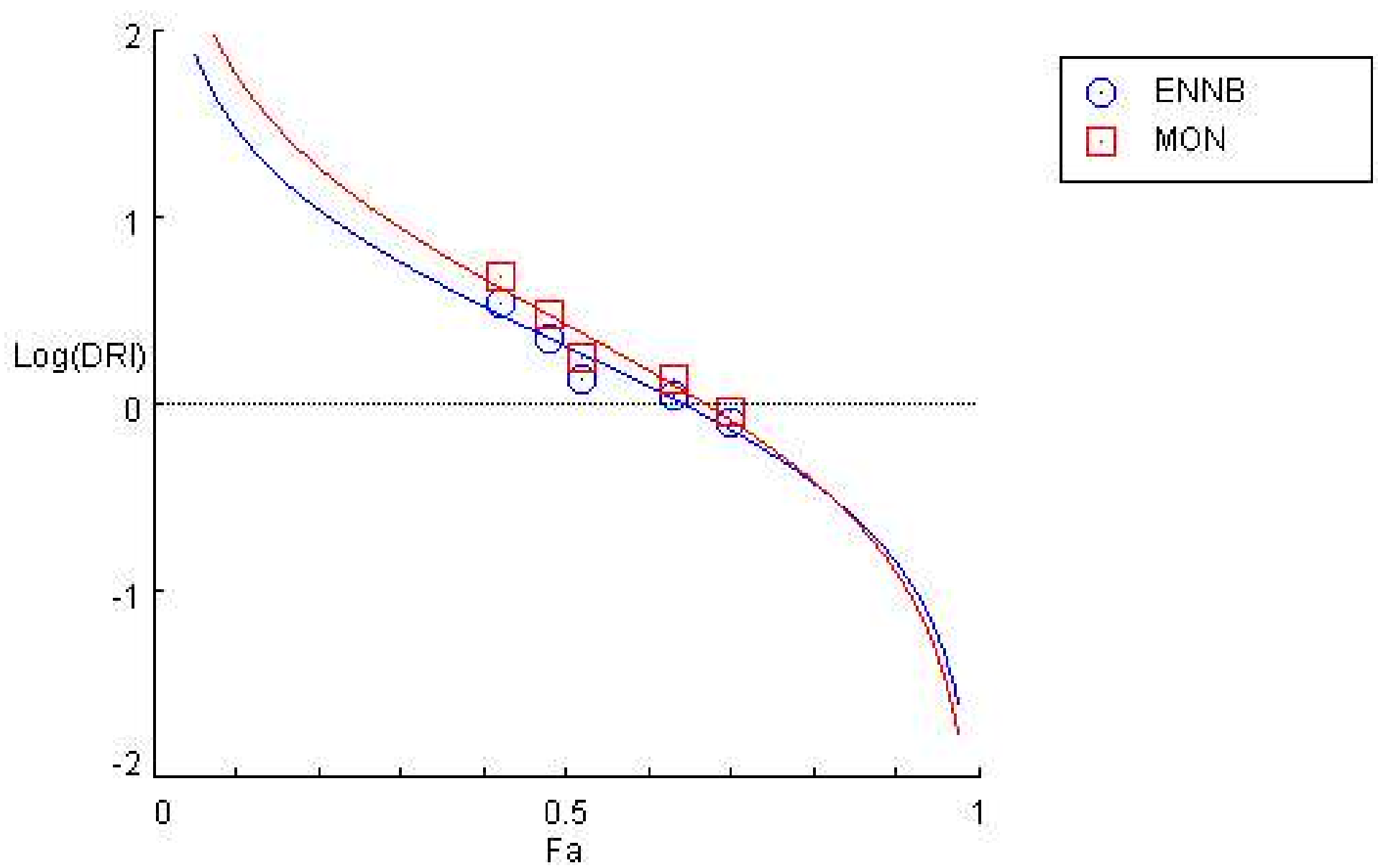

Log(DRI) Plot for Combo: BEENMO (BEA+ENNB+MON [0.45:1.12:10.11])

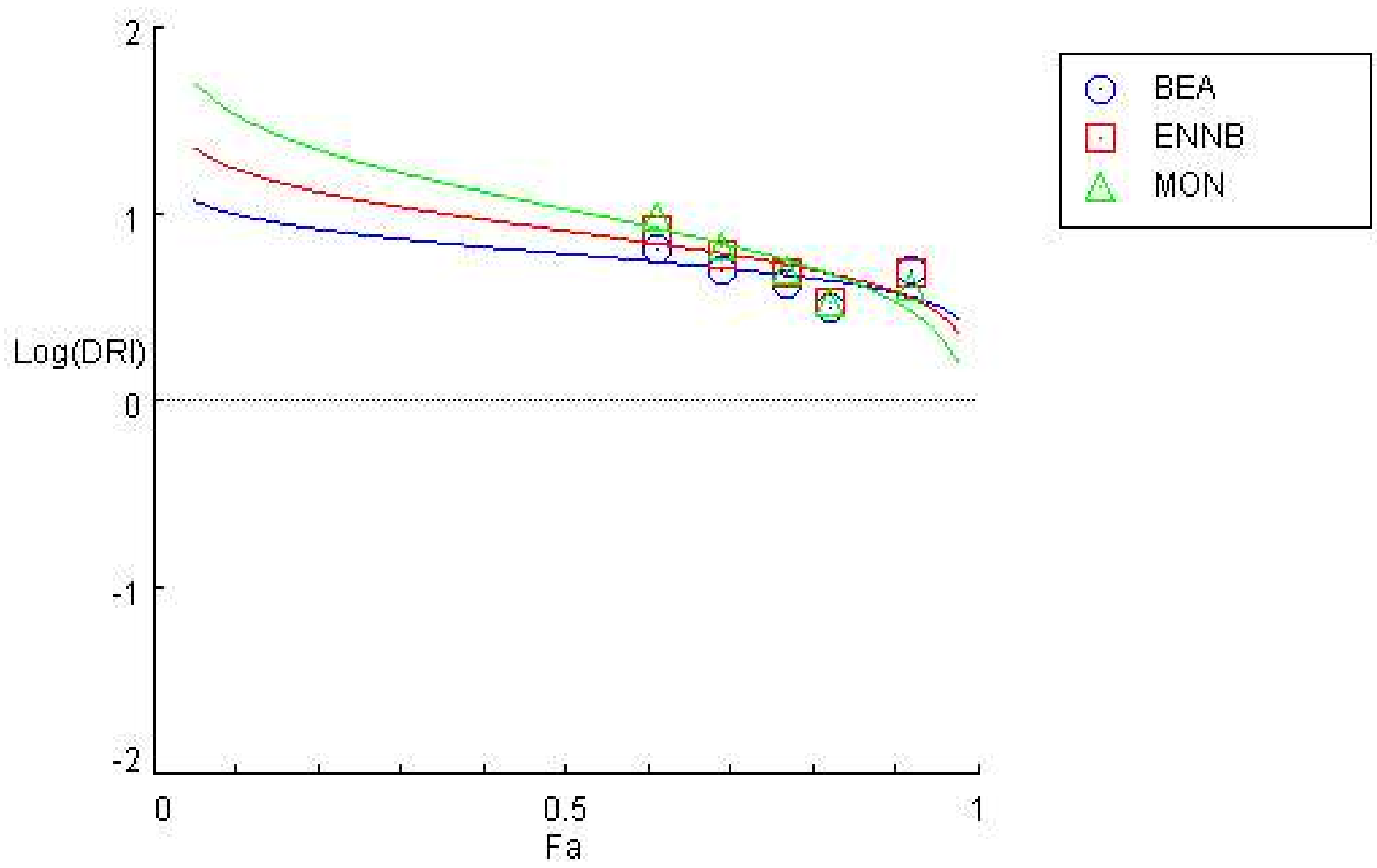

Isobologram for Combo: BEAENN (BEA+ENNB [0.45:1.12])

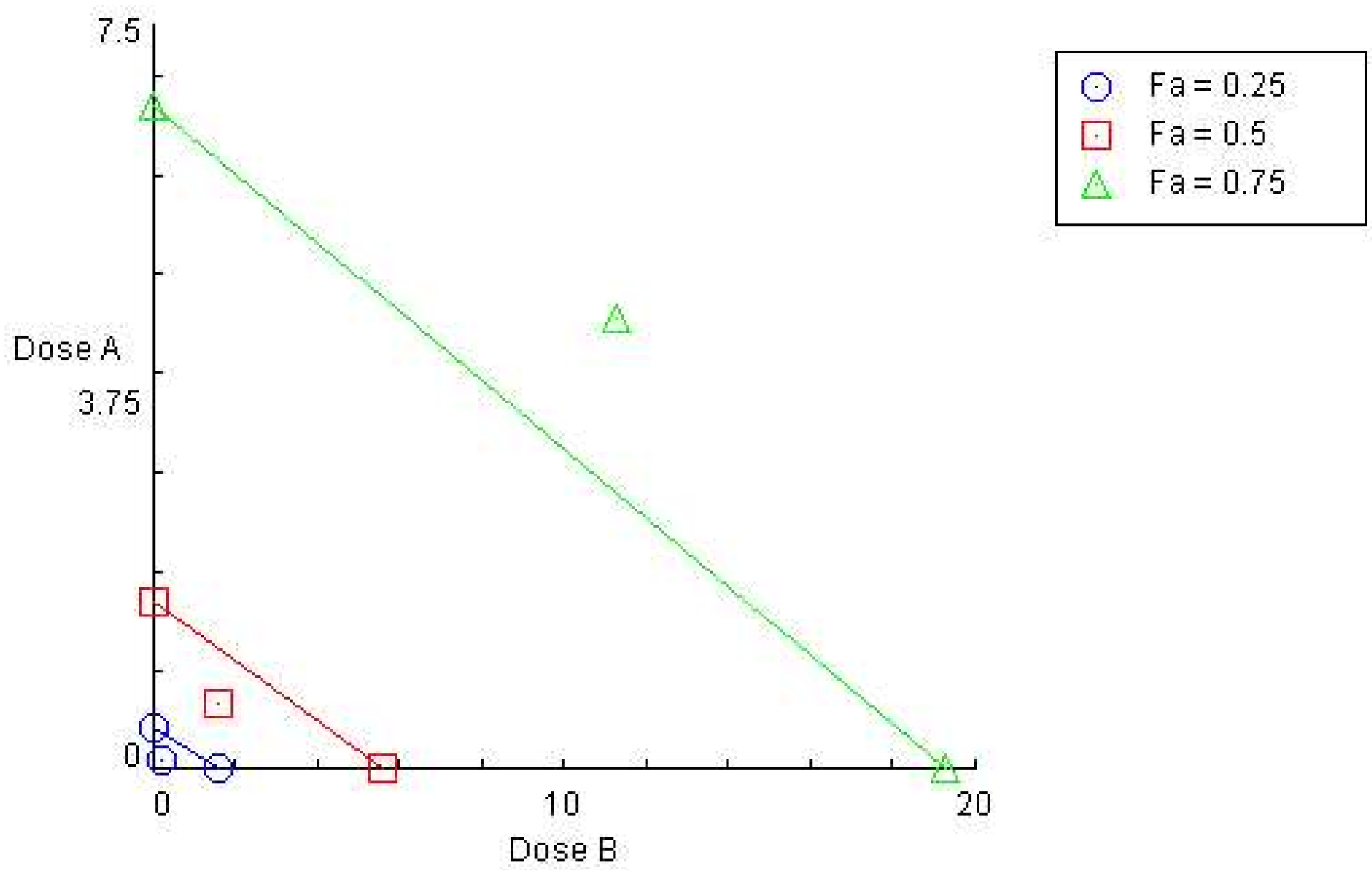

Isobologram for Combo: BEAMON (BEA+MON [0.45:10.11])

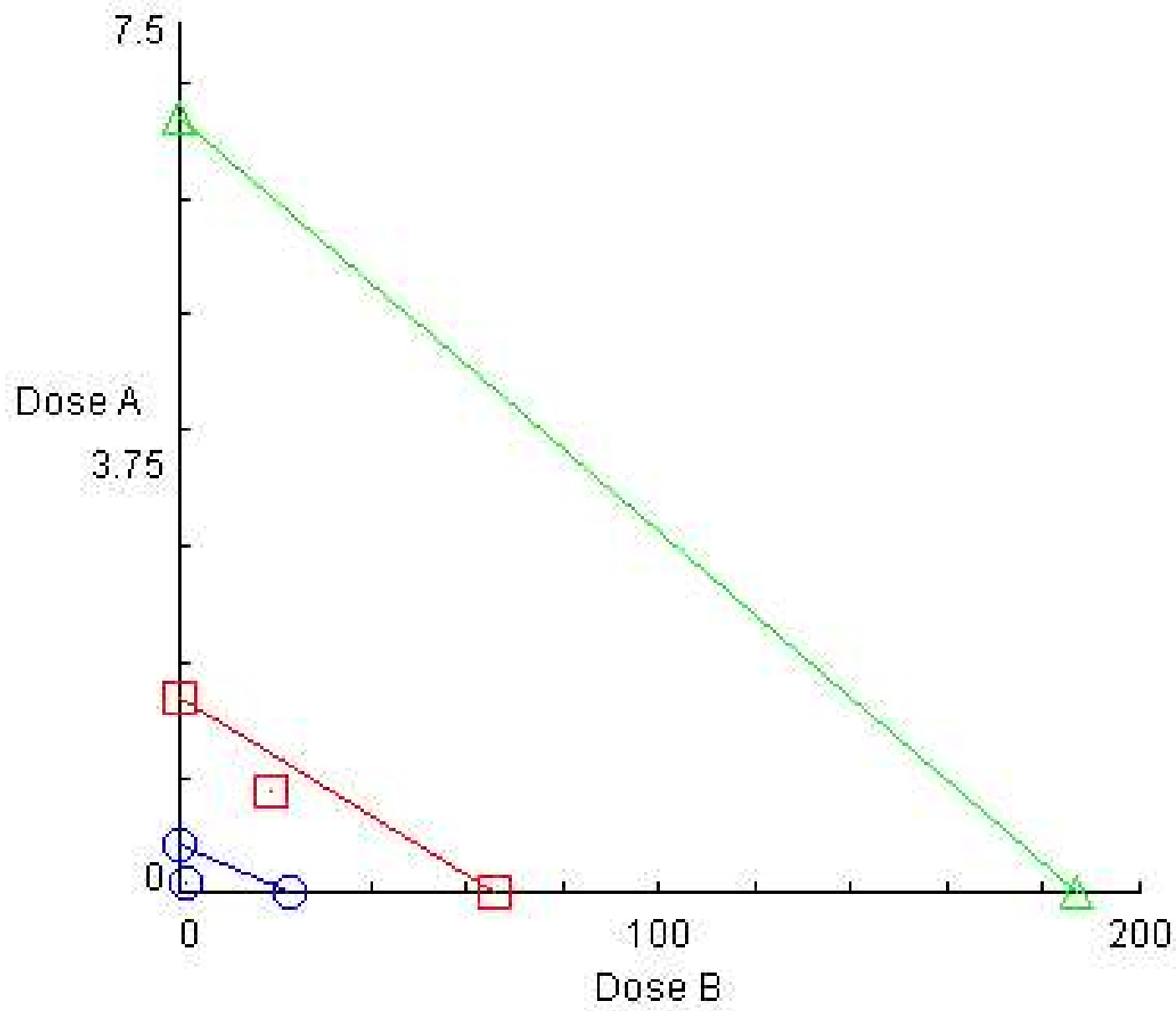

Isobologram for Combo: ENNMON (ENNB+MON [1.12:10.11])

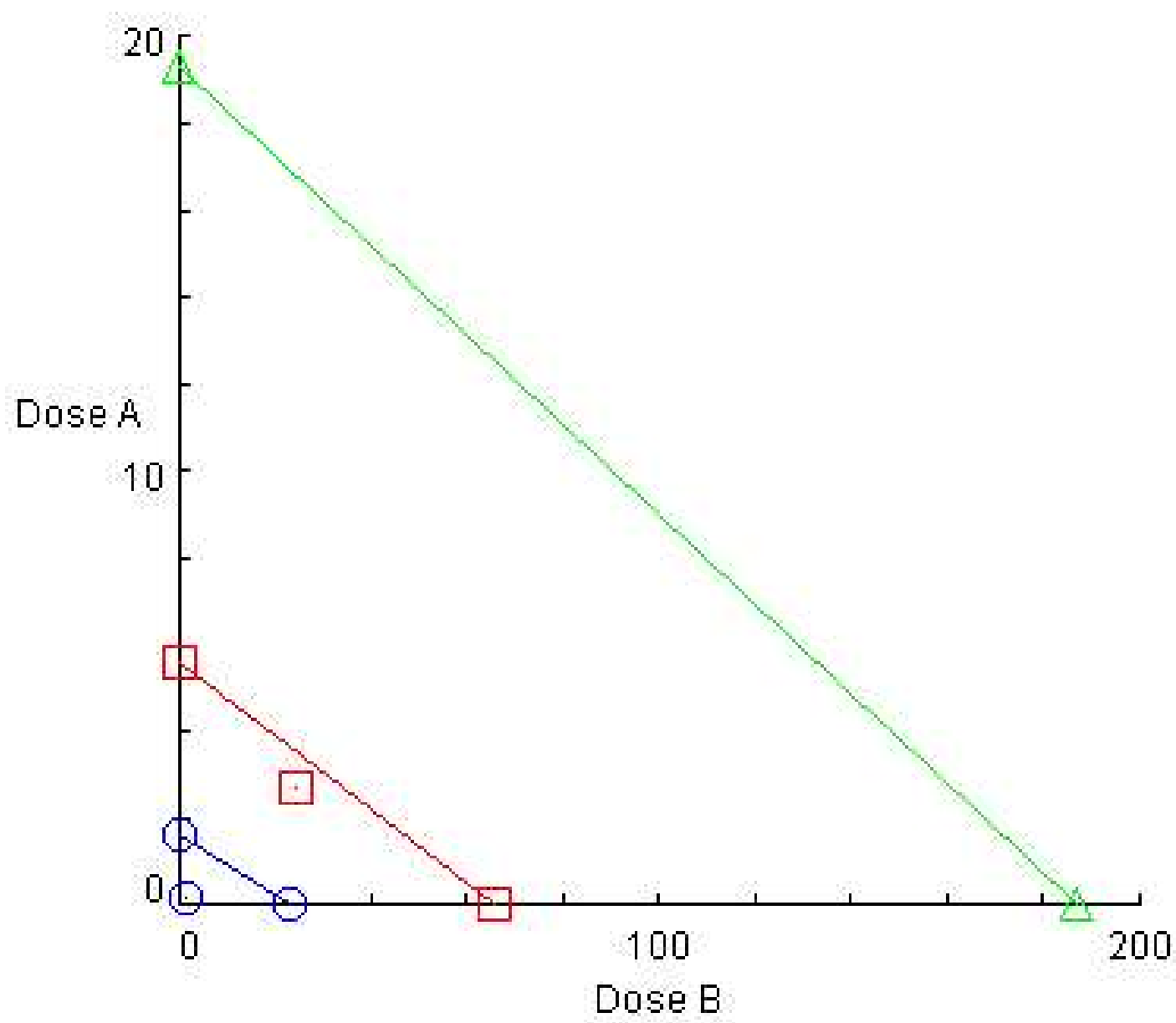

Polygonogram at Fa = 0.5

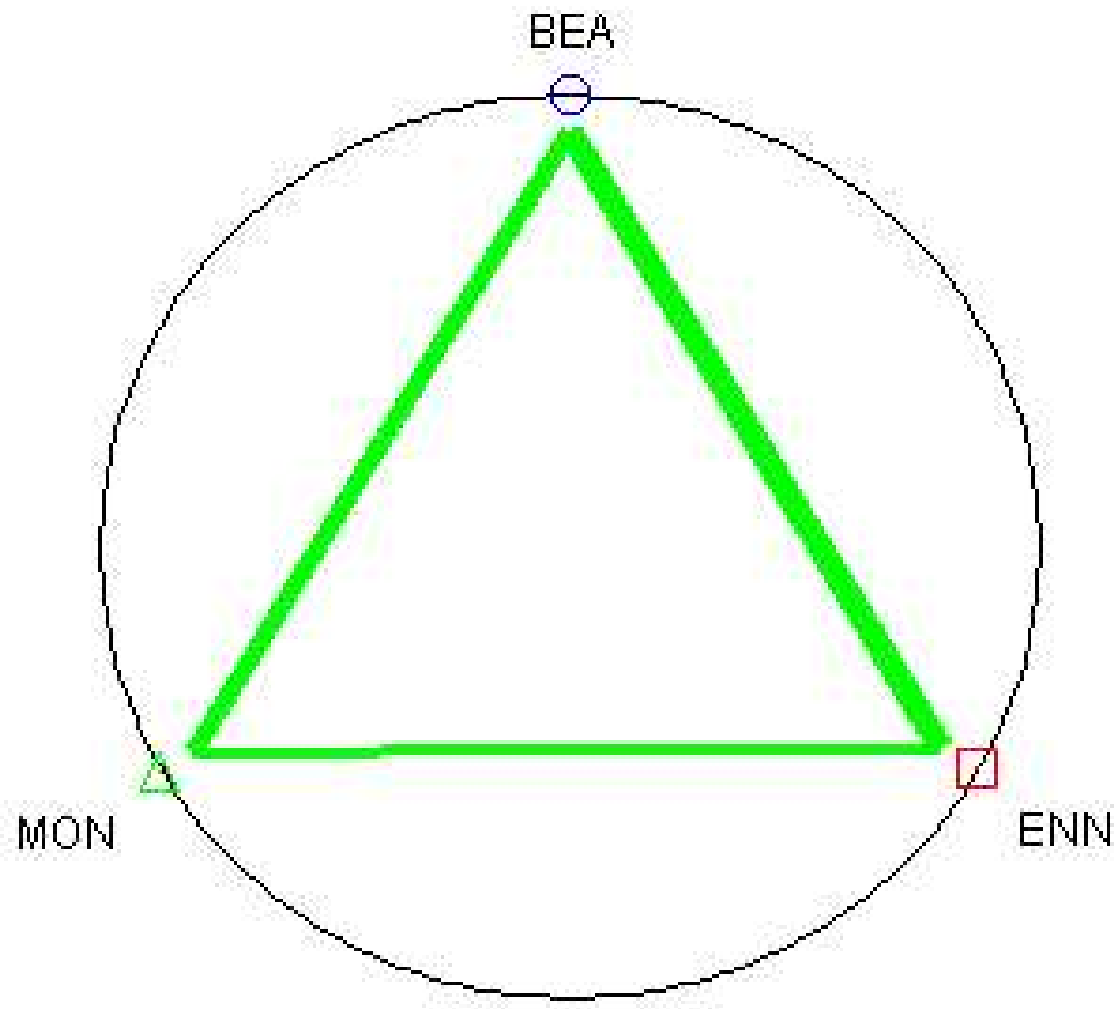

Summary Table

**Experiment Name:**	MTT BEA ENN MON
**Date:**	1.12.25
**File Name:**	C:\Users\user\OneDrive - Universite de Montreal\Documents\Postdoc\Poapolathep\2025\BEA ENN MON_01.12.25.cse
**Description**	

Drug: BEA (BEA) [µM]
Drug: ENN B (ENNB) [µM]
Drug: MON (MON) [µM]
Drug Combo: BEA-ENNB (BEAENN) (BEA+ENNB [0.45:1.12])
Drug Combo: BEA-MON (BEAMON) (BEA+MON [0.45:10.11])
Drug Combo: ENNB-MON (ENNMON) (ENNB+MON [1.12:10.11])
Drug Combo: BEA-ENNB-MON (BEENMO) (BEA+ENNB+MON [0.45:1.12:10.11])

**Drug/Combo**	**Dm**	**m**	**r**
BEA	1.69261	0.79796	0.98090
ENNB	5.59422	0.88645	0.99344
MON	66.0686	1.05557	0.94872
BEAENN	2.28692	0.56745	0.98331
BEAMON	20.5313	0.47175	0.99340
ENNMON	27.3267	0.42638	0.98704
BEENMO	7.08757	0.67829	0.97361

CI values at:

**Combo**	**ED50**	**ED75**	**ED90**	**ED95**
BEAENN	0.67889	1.26284	2.36012	3.62095
BEAMON	0.81441	2.41791	7.38064	16.0212
ENNMON	0.85954	3.58510	15.1010	40.3828
BEENMO	0.37567	0.54906	0.81776	1.08423

Data for Fa = 0.1

Drug/Combo	CI value	Dose BEA	Dose ENNB	Dose MON
BEA		0.10782		
ENNB			0.46910	
MON				8.24108
BEAENN	0.19892	0.01364	0.03396	
BEAMON	0.09963	0.00830		0.18653
ENNMON	0.05084		0.01576	0.14222
BEENMO	0.18519	0.01070	0.02663	0.24042

Data for Fa = 0.25

Drug/Combo	CI value	Dose BEA	Dose ENNB	Dose MON
BEA		0.42720		
ENNB			1.61996	
MON				23.3340
BEAENN	0.36666	0.09457	0.23537	
BEAMON	0.28156	0.08523		1.91480
ENNMON	0.20808		0.20722	1.87050
BEENMO	0.26163	0.05406	0.13454	1.21446

Data for Fa = 0.5

Drug/Combo	CI value	Dose BEA	Dose ENNB	Dose MON
BEA		1.69261		
ENNB			5.59422	
MON				66.0686
BEAENN	0.67889	0.65549	1.63144	
BEAMON	0.81441	0.87491		19.6563
ENNMON	0.85954		2.72537	24.6013
BEENMO	0.37567	0.27307	0.67963	6.13487

Data for Fa = 0.75

Drug/Combo	CI value	Dose BEA	Dose ENNB	Dose MON
BEA		6.70626		
ENNB			19.3186	
MON				187.068
BEAENN	1.26284	4.54344	11.3081	
BEAMON	2.41791	8.98139		201.782
ENNMON	3.58510		35.8447	323.563
BEENMO	0.54906	1.37939	3.43316	30.9904

Data for Fa = 0.9

Drug/Combo	CI value	Dose BEA	Dose ENNB	Dose MON
BEA		26.5707		
ENNB			66.7132	
MON				529.671
BEAENN	2.36012	31.4923	78.3810	
BEAMON	7.38064	92.1984		2071.39
ENNMON	15.1010		471.439	4255.58

Drug/Combo	CI value	Dose BEA	Dose ENNB	Dose MON
BEENMO	0.81776	6.96802	17.3426	156.548
